# Molecular Dynamics Simulation of Passive Diffusion across a Human Breast Cancer Cell Membrane Model. Comparison between Cisplatin and Its Pt(IV) Derivatives

**DOI:** 10.1021/acs.jcim.5c02819

**Published:** 2026-03-06

**Authors:** Daniele Belletto, Stefano Scoditti, Stefano Borocci, Nico Sanna, Costantino Zazza, Emilia Sicilia

**Affiliations:** † Department of Chemistry and Chemical Technologies, Università della Calabria, Ponte P. Bucci, Arcavacata di Rende, Calabria 87036, Italy; ‡ Department for Innovation in Biology Agro-Food and Forest Systems (DIBAF), University of Tuscia, Largo Dell’Università snc, Viterbo 01100, Italy

## Abstract

The efficacy of platinum­(II) drugs, despite their wide use in clinical practice, is seriously limited by their well-known drawbacks. Octahedral Pt­(IV) congeners are considered a sort of Holy Grail in cancer research as, being significantly more inert, they should be able to overcome the limitations of current platinum-based drugs, such as resistance and side effects, acting as prodrugs. Additionally, their anticancer activity can be tuned through a proper choice of the axial ligands released inside cancer cells when these compounds are reduced, making them even capable of potentially working as multiaction agents. However, despite their very satisfactory anticancer effects, no Pt­(IV) complex has been approved for clinical use. As cell membrane permeation is the critical step, very poorly understood, of the whole mechanism of action of any drug, the investigation of the eventual differences in behavior between four-coordinate Pt­(II) and six-coordinate Pt­(IV) complexes when they diffuse in a lipid bilayer might be of significant relevance. The outcomes of a biased molecular dynamics (MD) investigation of the permeation of cisplatin and three simple cisplatin Pt­(IV) derivatives through a membrane model prototype of human breast cancer cells are illustrated here. This comparative analysis of Pt­(II) and Pt­(IV) complex passive diffusion has been carried out with the aim of gaining indications about the factors that play a role in favoring or hindering membrane penetration and, ultimately, in determining the efficacy of their anticancer action.

## Graphical abstract

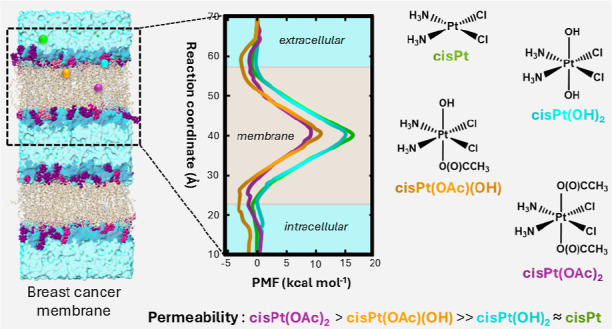

## 1 Introduction

The cytotoxic activity of the *cis*-diamminedichloridoplatinum­(II) complex (cisplatin, here cisPt) has been discovered fortuitously in 1965 when Barnett Rosenberg and co-workers examined the effect of electromagnetic fields on *Escherichia coli* cell division.^1^ The successful results of clinical trials led to the approval of the complex for the treatment of cancer by the Food and Drug Administration (FDA) in 1978 ^2^ and since then an enormous number of both experimental and theoretical papers dealing with the details of the cisplatin mechanism of action have been published.^3–12^ Based on the outcomes of these investigations, the proposed mechanism involves that in the cytosol the complex undergoes the hydrolysis of the Cl^–^ ligands that, due to the drastic decrease of chlorido ions’ concentration, are replaced by water molecules. Once hydrolyzed, the charged aquated complex can enter the cell nucleus and interact with DNA. In particular, the platinum center of the hydrolyzed drug undergoes a nucleophilic attack by the N7 atoms of the purine bases, mainly guanine, forming DNA adducts and cross-links that cause a distortion of the DNA helix and inhibit DNA replication and transcription.^6,8^ This damage triggers a DNA response that ultimately leads to apoptosis. Subsequently, various cisplatin structural analogues have been prepared and screened as potential antitumor agents,^13–15^ but only two of them, carboplatin, [*cis*-diammine­(1,1-cyclobutanedicarboxylato)­platinum­(II)][Bibr ref16] and oxaliplatin, [(1R,2Rcyclohexanediamine)­oxalatoplatinum­(II)][Bibr ref17] have been approved for their worldwide use as anticancer drugs. Unfortunately, a combination of several factors limits the efficacy of these Pt drugs: cellular drug resistance, toxicity, and poor pharmacokinetic profiles due to various factors such as upregulation of DNA repair pathways, low intracellular accumulation, inactivation by thiol-containing reductants, and inherently high reactivity that causes premature inactivation. One of the strategies viable for overcoming the typical drawbacks of cisplatin and its congeners is to use platinum­(IV) complexes, as pro-drugs.^18^ Pt­(IV) complexes, indeed, typically have low-spin d^6^ electronic configurations and exhibit an octahedral geometry. This configuration is relatively inert to substitution and reactions with biological nucleophiles and hydrolysis are thus disfavored compared to Pt­(II) complexes. As the lifetime in biological fluids is expected to increase Pt­(IV) prodrugs should reach cancer cells nearly intact. Even oral administration is feasible as degradation in the gastrointestinal tract is less likely.^19,20^ The critical step of the Pt­(IV) prodrug mechanism of action (MoA) is their reduction to the corresponding Pt­(II) cytotoxic counterparts. Reduction should take place prominently when they are internalized inside the cancer cell, where the right reducing environment exists created by high levels of cellular reductants handling the increased oxidative stress caused by reactive oxygen species (ROS) overproduction.^21^ The nature of the axial and, to a lesser extent, equatorial ligands is decisive in determining their propensity to undergo two-electron reduction, which provokes the breaking of the bonds between the Pt center and axial ligands. A careful choice of the ligands, therefore, allows us to avoid that the reduction occurs too rapidly before the prodrug reaches the tumor or too slowly if the prodrug resists the action of the reducing agents. Additionally, axial ligands can be utilized to enhance the pharmacological properties of the prodrug, selecting ligands able to target cancer cells and/or to facilitate cell uptake. Indeed, drugs’ permeation across cell membranes is a crucial step, very poorly understood, of the whole MoA that largely determines the efficacy of the drugs.^22^ Therefore, even if efficient uptake is not a synonym of the efficacy of a drug action, it is a necessary precondition. Although membrane transporters, in particular copper Ctr1 ones, are involved,^23,24^ the most viable pathway for intracellular accumulation of Pt drugs is passive diffusion driven by gradient concentration through the plasma.^25,26^ On the basis of such premises, we have simulated by means of biased Molecular Dynamics (MD) the permeation of the parent cisplatin drug and three simple cisplatin Pt­(IV) derivatives (see Scheme 1) through the membrane model adopted in a previous computational study dealing with the passive diffusion process of Pt­(II)-based drugs across a realistic plasma membrane prototype of human breast cancer cells.^27^ Cytotoxicity of the three cisplatin Pt­(IV) derivatives: cisPt­(OH)_2_ having two hydroxido ligands in axial position, cisPt­(OAc)­(OH) having an acetate and a hydroxido and cisPt­(OAc)_2_ having two acetates, has been intensively investigated,^28^ highlighting the higher cytotoxicity of the cisPt­(OAc)­(OH) complex as a consequence of its peculiar cellular accumulation. Such peculiar accumulation has been ascribed to the involvement of an assisted transport that is complementary to or alternative to passive diffusion. Potential of mean force (PMF) computed for all of the investigated complexes along the permeation pathway has been used, together with the analysis of the change of various structural and chemical–physical membrane parameters, as the key tool for describing the details of the transfer process from bulk water to the membrane interior. This comparative investigation of Pt­(II) and Pt­(IV) complex passive diffusion, to the best of our knowledge the first concerning Pt­(IV) derivatives, aims at highlighting the expected differences in behavior and extracting indications about the impact that such differences, if they exist, might have on the efficacy of the examined drugs and their future design.

**Scheme 1:**
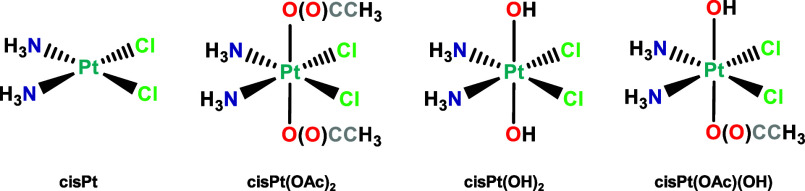
Schematic sepresentation of the structure of the Pt complexes under investigation.

## 2 Results and Discussion

### 2.1 Cisplatin Permeation

This section is dedicated to the description of the potential of the mean force free energy profile describing the cell permeation of cisPt, as the reference compound, in comparison with previous investigations and to the outline of the results of the analyses carried out for rationalizing the observed behavior. Further comments about the calculated PMF profiles and the corresponding description are available in Figure S1 of the Supporting Information. In addition, in order to confirm the exact position of the four complexes at the interfaces and at the membrane bilayer core, electron density profiles have been reported in the Supporting Information (Figure S2).

#### 2.1.1 Cisplatin PMF Profile

Several MD studies on the passive diffusion of cisPt across lipid bilayers of cell membrane models of various compositions, providing detailed information about the interactions and factors that affect its uptake, already exist in the literature.^27,29–33^ In spite of the different adopted simulation conditions, including the composition of the membrane models, common characteristics of all the reported PMF profiles can be highlighted. Favorable interactions are established by the drug with the polar heads at the water–membrane interface when the complex leaves the water environment. The largest stabilization has been calculated by Almeida *et al.*, being the well 8.6 kcal mol^–1^ deep. Rivel *et al.* calculated a free-energy stabilization of 5.6 kcal mol^–1^ and, additionally, a very small energy barrier of only 0.2 kcal mol^–1^ in the region of polar heads is reported in their study. An analogous very small free-energy barrier of about 0.2 kcal mol^–1^ has been intercepted also by Nogueira and co-workers,^29^ while a free-energy minimum of 0.65 kcal mol^–1^ has been predicted at the entrance of the polar region of the bilayer. An exception is the MD analysis carried out by Nierzwicki *et al.* ^30^ whose outcomes do not show any preference of cisPt for residing in the polar headgroup region than in the water phase. A pronounced energy barrier for the permeation process through the lipid tails has been intercepted by all of the previous investigations. Almeida *et al.* ^27^ reported a very high energy barrier of about 28.0 kcal mol^–1^ calculated with respect to the previous minimum. A free energy increase of 10.4 kcal mol^–1^ with respect to the energy of the global minimum has been calculated by Nogueira and co-workers^29^ in agreement with the values of previous MD calculations: 12.0 and 9.6 kcal mol^–1^ by Nierzwicki *et al.* ^30^ and Yesylevskyy *et al.*,^33^ respectively. Values of the barrier as a function of the lipid asymmetry and cholesterol content have been calculated by Rivel et al.,^32^ going from 9.6 kcal mol^–1^ for a pure DOPC membrane up to 16.7 kcal mol^–1^, increasing the cholesterol content from 0% to 33%. A study by the same author of the cisPt permeability as a function of the membrane curvature has shown an increase in permeability upon membrane bending, with the height of the barrier decreasing from 16.8 kcal mol^–1^ for the flat membrane to 15.1 kcal mol^–1^ for the convex outer layer.

The symmetric permeation free energy profiles for the selected drugs cisPt and its three Pt­(IV) derivatives, simulated using a realistic plasma membrane model reproducing human breast cancer cells, is shown in Figure 1, while Table 1 summarizes the most relevant information extracted from the PMF profiles for the four complexes. Inspection of Figure 1 panel A shows that when the cisPt drug is pulled from the right side of the symmetric membrane from bulk water to the polar headgroup region, no resistance is opposed to the permeation at the water–lipid interface. While proceeding deeper in the bilayer, the complex displays only a slight preference for the polar headgroup region compared to the water phase for a decrease in free energy of only 0.8 kcal mol^–1^ at a distance of 57.3 Å from the COM. The height of the barrier calculated with respect to the previous minimum for the crossing of the interleaflet regions is 16.8 kcal mol^–1^, which clearly indicates that the metallodrug actually experiences repulsive interactions when it continues the permeation through the lipid tails and reaches the center of the membrane. As illustrated in the next paragraphs, the drug experiences different interactions at the interface located at about 23 Å from the COM on the left side of the membrane. Despite this difference, the slightly favorable attraction between the drug and the polar headgroup region of the bilayer corresponds to a free-energy minimum at 23 Å having a depth, with respect to the energy of cisplatin located in the bulk solvent, of 0.8 kcal mol^–1^ that mirrors that at the opposite interface.

**Figure 1:**
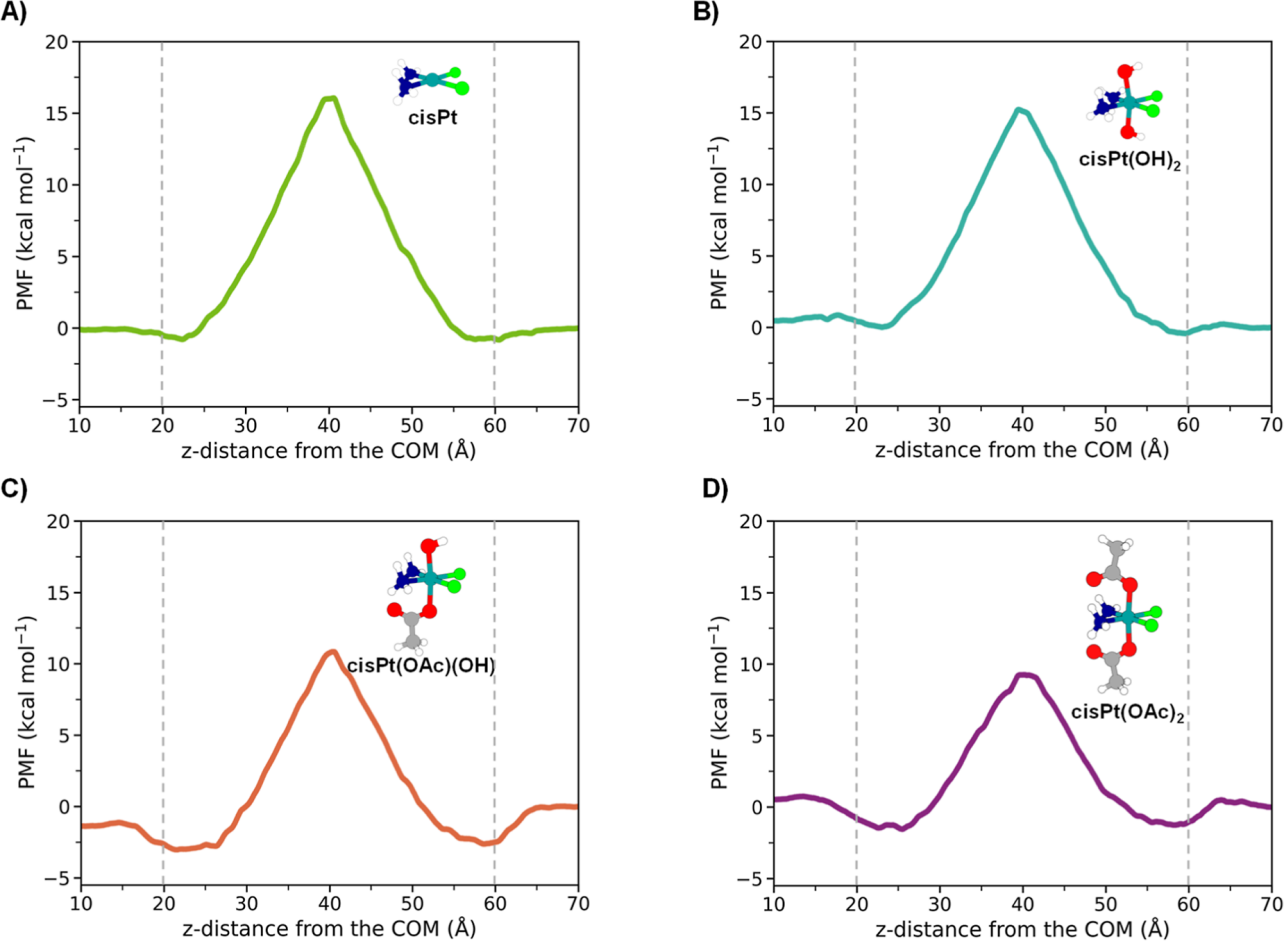
Estimated PMF profiles calculated for (A) cisPt, (B) cisPt­(OH)_2_, (C) cisPt­(OAc)­(OH), and (D) cisPt­(OAc)_2_, permeating the adopted symmetric model membrane. The dashed vertical gray lines indicate the position of the lipid headgroups.

**I tbl1:** Energy values, expressed in kcal mol^–1^, extracted from the PMF profiles for the four complexes^a^

	**Min_right_**	**Max**	**Min_left_**	**ΔG_right_**	**ΔG_left_**
**cisPt**	–0.8 (57.3 Å)	16.0 (40.0 Å)	–0.8 (22.4 Å)	16.8	16.8
**cisPt(OH)_2_**	–0.5 (59.4 Å)	15.2 (39.5 Å)	0.0 (23.0 Å)	15.8	15.2
**cisPt(OAc)(OH)**	–2.7 (58.6 Å)	10.8 (40.4 Å)	–3.1 (21.5 Å)	13.5	14.0
**cisPt(OAc)_2_**	–1.3 (58.2 Å)	9.2 (40.5 Å)	–1.6 (25.4 Å)	10.5	10.7

a Positions, expressed in Å, at the interfaces (**Min_right_** and **Min_left_**) and at the membrane bilayer core (**Max**) are reported in brackets with respect to the COM. The statistical uncertainties estimated from bootstrap analysis are below 0.01 kcal mol^‑1^ for all reported points and therefore are omitted.

#### 2.1.2 Interaction of cisPt with the Membrane Bilayer. Analysis of Contacts, Hydrogen Bonds, MM-GBSA, and Hydration

##### 2.1.2.1 Analysis of Contacts

The contact analysis of cisPt with water and lipid components at different *z*-distances from the center of the whole system reveals a clear evolution of its interaction profile as the drug moves from the bulk aqueous phase toward the bilayer interior (Figure S3). In bulk water, 70 Å from the COM of the entire system, cisPt exhibits 417 non-native contacts with water molecules at an average distance of 1.9 Å, indicating a highly dynamic hydration shell typical of small and polar compounds in aqueous solution. Only one native water molecule persists, suggesting a weakly structured hydration environment with rapid molecules exchange. Contacts with lipid headgroups, in particular, of PC, are negligible (24 non-native contracts at 4.2 Å), while contacts with other lipids are absent, consistently with cisPt positioned far from the membrane surface.

At the interface, at about 58 Å from the COM, cisPt begins to interact significantly with lipid headgroups and interfacial water. Notably, it forms 35 native contacts with PC at 2.0 Å and 10 native contacts with OL at 2.4 Å, indicating the emergence of more persistent interactions with PC and atoms in the upper part of the lipid tails. Water contacts decrease sharply, being only 2 native and 162 non-native at 2.0 Å, according to the partial insertion of the drug into the interfacial region, where the water accessibility drops even if remaining still substantial. Contacts with other lipid heads, such as PE and PS, are rare and occur at longer distances (4.5–6.0 Å), indicating weaker or transient interactions. Deeper inside the membrane, at about 40 Å from the COM, cisPt shows a dramatic increase in native contacts with water molecules (63 native) at a short distance (2.0 Å), while the total number of nearby water molecules is reduced, suggesting that cisPt drags water into the membrane interior, forming a sort of small water pocket which persists within the bilayer. Contacts with CHL and OL are also observed, implying that cisPt interacts directly with the hydrophobic core components, perhaps via hydrogen bonds to polar sites or via interaction with oxygen atoms of the lipids. At the opposite interface, located about 23 Å from the COM, cisPt regains stronger contacts with PC and PE lipid heads and water. In particular, it establishes 39 native contacts with PE at about 2.6 Å, 20 native contacts with PC at 2.5 Å, and 3 native contacts with water at 2.0 Å. The contact pattern resembles that at the interface with the extracellular environment, but with more native interactions with PE. Despite this difference and the highlighted asymmetric behavior at the interfaces, the stabilization is the same, as suggested by the symmetric PMF profile (Figure 1A). It is worth mentioning that contacts with CHL remain substantial, indicating persistent association with CHL rich regions, even at the interface.

Based on these observations, it is possible to conclude that while in bulk water cisPt is fully solvated with rapid water exchange, once it approaches and enters the membrane, the hydration shell diminishes and becomes more structured, with fewer but more persistent water molecules around it. The gradual increase in native contacts with lipid groups such as PC and PE lipid heads and OL tails reflects the stepwise insertion process during which cisPt engages first with polar heads at the interface and later with more polar regions within the bilayer core. Strong contacts with CHL and OL in the interior part of the bilayer suggest that cisPt interacts preferentially with certain lipid species, which probably stabilize its insertion path. Overall, the contact analysis reveals that cisPt during the transition from a fully hydrated state in bulk water to partially dehydrated and lipidic phases within the bilayer forms persistent contacts with phospholipid headgroups and CHL as it penetrates the membrane while maintaining a small but structured hydration shell throughout.

##### 2.1.2.2 Hydrogen Bonds

The hydrogen bond analysis provides an indication of how the complex interacts with its surrounding environment, reinforcing the evidence previously reported through contact analysis. Hydrogen bonds in bulk water are established by cisPt with water molecules; while moving toward the interface region, the local environment becomes increasingly heterogeneous, and the complex begins to interact not only with various lipid heads but also occasionally with the upper portion of lipid tails. However, despite these new contacts, water molecules remain the primary partners for hydrogen bonding. Deeper within the hydrophobic bilayer core, where the surroundings are mostly nonpolar, hydrogen bonds become less frequent and short-lived, typically formed with CHL and OL tails. Interestingly cisPt is capable of dragging water molecules into the center of the bilayer, making it less hydrophobic, as confirmed by water hydrogen bonds detection. Upon reaching the opposite side of the membrane, at about 23 Å from the COM, the number of hydrogen bonds with water increases once again, maintaining some contacts with CHL or OL while also establishing new hydrogen bonds with polar heads. As underscored in the previous paragraph, cisPt displays a highly dynamic hydration shell, primarily behaving as a hydrogen bond acceptor.

##### 2.1.2.3 MM-GBSA

The MM-GBSA approach was adopted to study the affinity between the membrane model and cisPt and its Pt­(IV) derivatives and assess the role of each term contributing to the total energy considering three key positions, about 58 (**Min_right_**) and 23 Å (**Min_left_**) and 40 Å (**Max**), for the translocation process. The values of the single terms of the total energy for all the four investigated complexes in correspondence of the interfaces and core positions are collected in Table S1 of the Supporting Information. CisPt membrane penetration is weakly unfavorable in the bulk water solvent (70 Å), establishing marginal or slightly unfavorable bonds, as attested by the small positive value, 0.4 kcal mol^–1^, of Δ*G*_total_ that becomes more favorable moving toward the two interfaces while it decreases again at the interleaflet region. Δ*E*_vdW_ contribution represents the driving force, becoming substantially more negative as the complex moves into the membrane, especially in correspondence of **Min_right_** and **Min_left_** interfaces. The electrostatic contribution, Δ*E*_ele_, is also highly stabilizing for the membrane-complex system due to the interactions established by the drug with membrane constituent lipid charges and/or polar groups, which drive membrane penetration. These interactions, as expected, are more favorable at the minima, especially at the second interface, as the drug is closer to the polar part of the bilayer and the aqueous environment, while their strength significantly decreases at the COM, where the drug is in contact with the apolar ends of the lipid tails.

##### 2.1.2.4 CisPt Hydration

A comparison between the profiles reporting the number of water molecules surrounding the four Pt complexes, calculated using a distance threshold of 3 Å from the ligand, as a function of their positions in the membrane, is reported in Figure 2. The number of water molecules in the first solvation sphere is 13 when cisPt is in the bulk solvent (panel A of Figure 2), in qualitative agreement with previous studies^29^ adopting different computational protocols. CisPt is expected to be surrounded by fewer water molecules in the hydrophilic region of the membrane than in bulk water. Indeed, about 9 molecules are lost from the hydration sphere in correspondence to the interface region on the right. This number slightly increases as cisPt moves toward the hydrophobic core, and when the ligand progresses through the hydrophobic tail region, water molecules are retained and there are no instances of complete dehydration. This behavior is reproduced in the left part of the profile. Additionally, Figure S4 of the Supporting Information shows the graphs of the radial distribution function, *g*(*r*), for cisPt and its Pt­(IV) derivatives in water at the three key positions, 58 and 23 Å (interfaces) and 40 Å (core), of the PMF profile.

**Figure 2:**
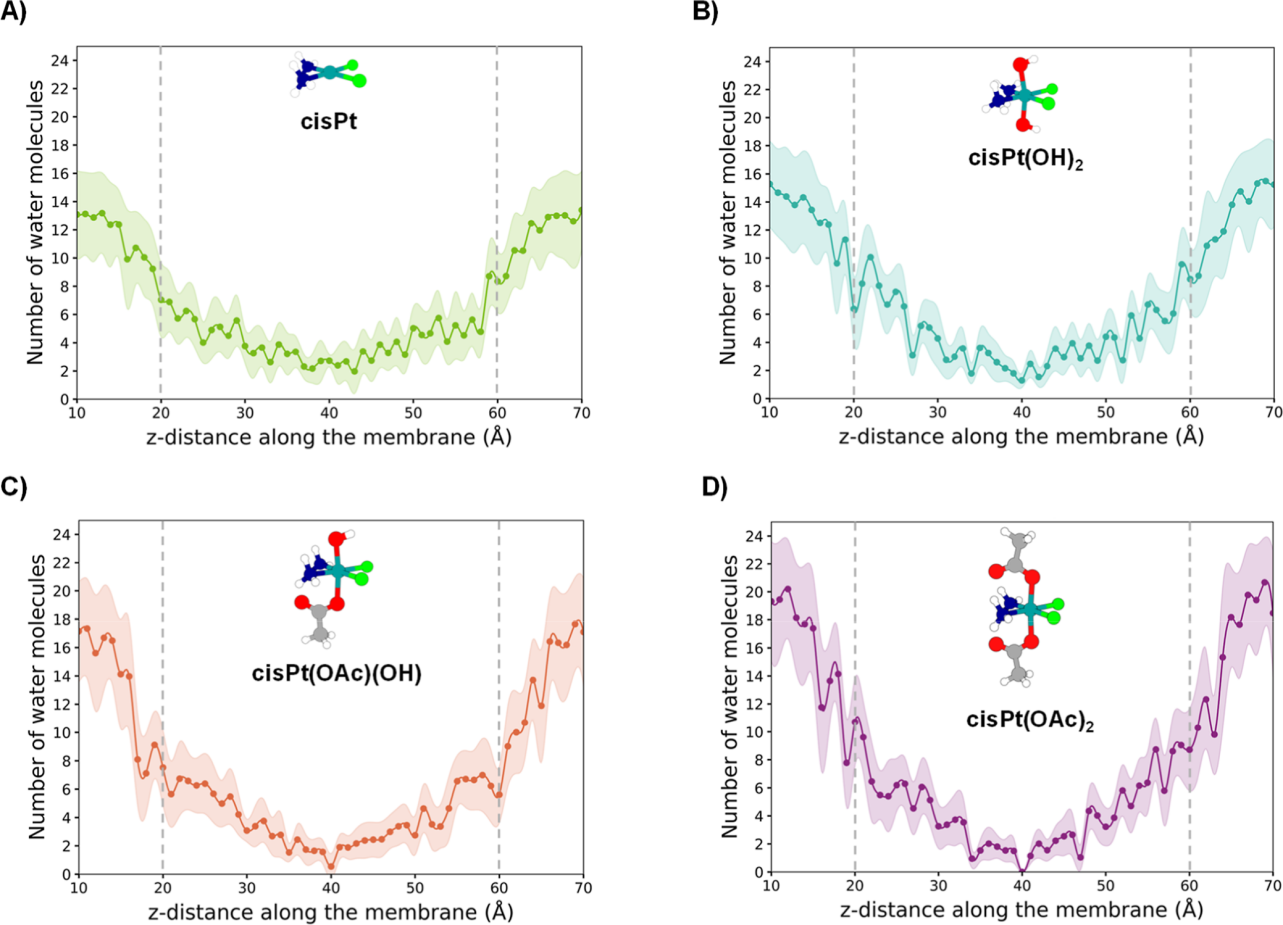
Profiles reporting the number of water molecules surrounding (A) cisPt, (B) cisPt­(OH)_2_, (C) cisPt­(OAc)­(OH), and (D) cisPt­(OAc)_2_, along the permeation path from the extracellular to the intracellular environment. The shaded regions correspond to the ± 1σ of the water shell population computed from frame-by-frame fluctuations within each umbrella sampling window, reflecting the fluctuations of the water shell within each window.

##### 2.1.2.5 CisPt Diffusion, Permeability, and Resistance

In the inhomogeneous solubility-diffusion framework, the permeability coefficient, P, arises from the integration of the position-dependent diffusivity, D­(*z*), and the potential of mean force (PMF), G­(*z*), along membrane normal *z* (see eq 1 in the Methods section). The exponential dependence on the free energy profile G­(*z*) reflects the thermodynamics of partitioning and barrier crossing, while the diffusivity coefficient D­(*z*), which P inversely depends on, describes how fast a molecule moves at different locations (*z*) across the membrane. For this reason, changes in the PMF can dominate the overall permeability response even when diffusivity differences are not negligible.^34^

Common characteristics of the four complexes are the higher diffusivity near the aqueous bulk on both ends of the membrane coordinate, in the range between about 0.8 and 1.4 × 10^–5^ cm^2^ s^–1^, consistent with their free mobility in water. They differ in bulk diffusion as attested by the different magnitudes, which can be related to physical–chemical properties such as size, polarity, and hydration. Diffusion drops sharply moving from bulk water to the headgroup region, due to both steric hindrance and energetic barriers imposed by the transition from the aqueous to the amphiphilic environment. In correspondence of the interfaces, there are minima in D­(*z*) values (Table S2) because interactions with polar and charged groups of lipid heads, together with water molecules that still surround the complexes, slow down translational motion. Near the bilayer midplane, the D­(*z*) values slightly increase, still remaining lower than those in bulk water. This is the same point where resistivity R­(*z*) has a peak (Figure S5) because the nonpolar core is the most energetically unfavorable region for polar drug diffusion. The magnitude and width of these R­(*z*) vary significantly across complexes.

In a specific manner, the precursor cisPt experiences a moderate reduction of D­(*z*) in the core (Figure 3), but not as low as expected for larger hydrophilic species, while R­(*z*) shows a sharp, high peak in correspondence of the center of the double layer, indicative of a distinct free-energy barrier for crossing the hydrophobic core and in line with the previously reported PMF profile. These findings are in line with other MD studies showing that cisplatin can spontaneously enter membranes, but faces a significant barrier in the tail region.^27,29,30,32,33^

**Figure 3:**
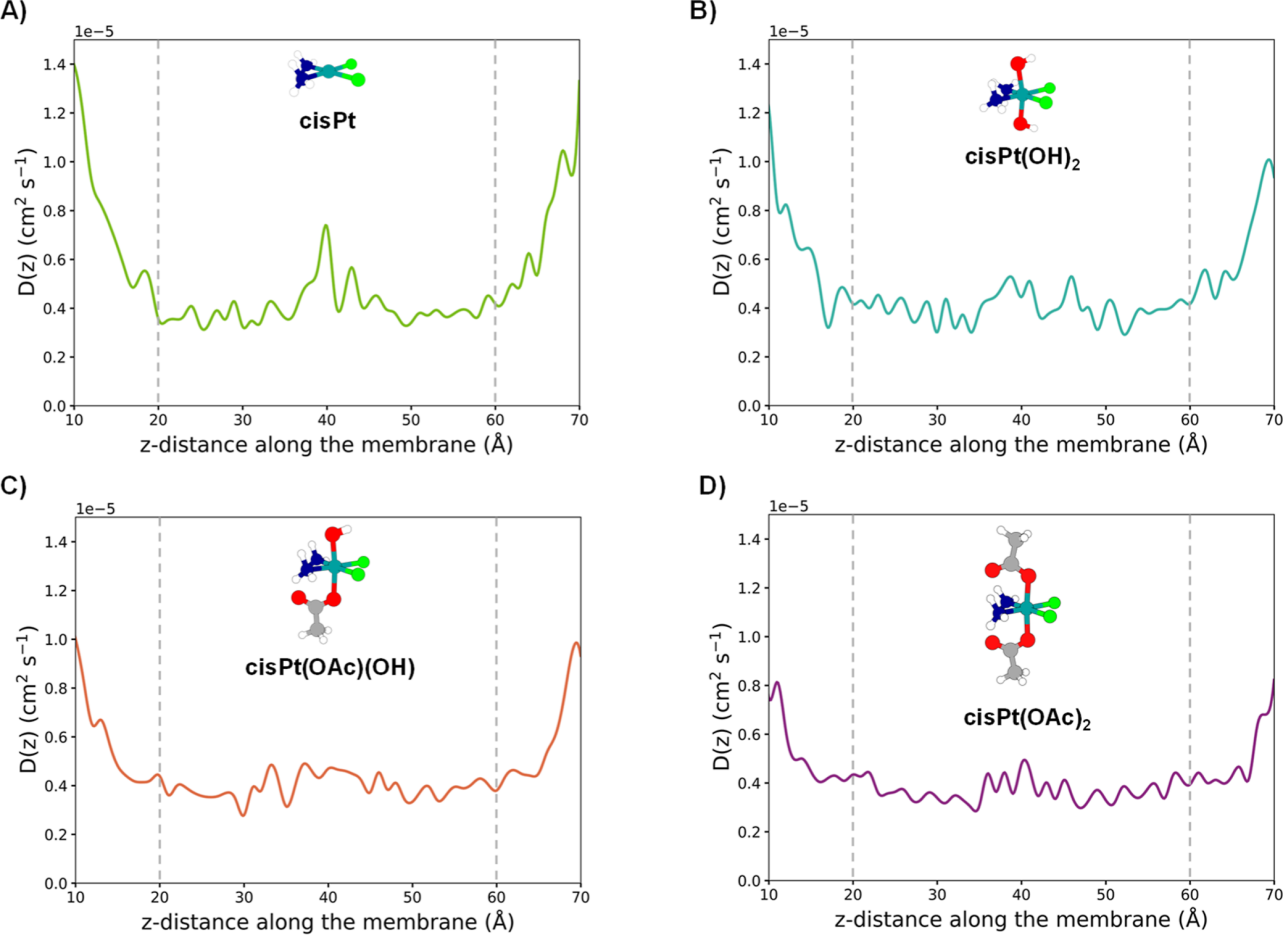
Diffusion profiles of (A) cisPt, (B) cisPt­(OH)_2_, (C) cisPt­(OAc)­(OH), and (D) cisPt­(OAc)_2_, along the permeation path from the extracellular to the intracellular environment. The dashed vertical gray lines indicate the position of the lipid headgroups.

The computed *P* and *R*_eff_ values are listed in Table II. The highest *R*_eff_ for cisPt (6.7 × 10^9^) reflects the largest central membrane barrier, in line with small, neutral but highly hydrated species that lose water partially entering the hydrophobic core. It faces a significant solubility penalty in the lipid tail region. The permeability value for cisPt calculated by us has been compared with that obtained using an asymmetric membrane model to mimic cancer cells including in vitro data.^32^

### 2.2 Permeation of Pt­(IV) Complexes

The key characteristics of the transport of the six-coordinate cisPt­(OH)_2_, cisPt­(OAc)­(OH), and cisPt­(OAc)_2_ complexes through the adopted cell membrane model are captured by PMF profiles and the outcomes of a series of analyses that are compared with those of the four-coordinate cisPt complex. Further comments about the PMF profiles can be found in the Supporting Information (Figure S1 and the corresponding description), together with electron density profiles confirming the exact position of each complex at the interfaces and at the membrane bilayer core (Figure S2).

#### 2.2.1 PMF Profiles of Pt­(IV) Complexes

The PMF profiles describing the passive translocation of the three Pt­(IV) complexes are reported in panels B–D of Figure 1 for cisPt­(OH)_2_, cisPt­(OAc)­(OH), and cisPt­(OAc)_2_, respectively. As it clearly appears, the first complex having two hydroxido ligands in axial position shows a behavior very similar to that of the cisPt precursor. The interaction of the drug with the polar region of this lipid bilayer, at about 57.2 Å from the COM, is only slightly favorable, causing an energy drop of only 0.5 kcal mol^–1^ (see Table 1) and no energy barrier hampers the permeation at the water–lipid interface. Continuing the permeation of the drug through the membrane, the process, as predictable, becomes even less favorable because of the van der Waals interactions established with the lipid tails, which correspond to an energy increase that accompanies the diffusion of the drug until it reaches the center of the membrane. The height of the estimated barrier is 15.8 kcal mol^–1^, calculated with respect to the previous shallow minimum. On the left side of the membrane at the interface, at about 23 Å from the COM, the pathway is almost flat, and no favorable interaction is established by the drug with the polar region of the leaflet with respect to the bulk water of the intracellular environment. This behavior is, very likely, a consequence of the hydration shell that, as illustrated below, surrounds and accompanies the drug during the permeation process.

**II:** Permeability (P) and effective resistance (*R*_eff_) values, expressed in cm s^–1^ and in s cm^–1^, respectively, extracted from the diffusion profiles of the four complexes across the membrane model.

	***P***	***R*_eff_**
**cisPt**	1.5 × 10^–9^	6.7 × 10^9^
	6.57 × 10^–9^ ^a^	
**cisPt(OH)_2_**	4.7 × 10^–9^	2.1 × 10^8^
**cisPt(OAc)(OH)**	5.6 × 10^–6^	1.8 × 10^5^
**cisPt(OAc)_2_**	4.1 × 10^–5^	2.5 × 10^4^

a see ref 32.

The behaviors of the asymmetric complex cisPt­(OAc)­(OH) and the symmetric one cisPt­(OAc)_2_ are described based on the PMF profiles reported in Figure 1 panels C and D, respectively. The cisPt­(OAc)­(OH) drug, having a polar OH ligand and a more lipophilic acetate one, in the transition from the extracellular water environment to the interface (at 58.6 Å) with the bilayer, establishes stabilizing interactions that result in a free energy minimum of −2.7 kcal mol^–1^ with respect to the entrance. A minimum at 58.2 Å, having a depth of 1.3 kcal mol^–1^, characterizes the permeation profile of the cisPt­(OAc)_2_ complex. No free energy increase accompanying the entrance of the drugs in the polar region is registered. As the two complexes continue to diffuse toward the center of the bilayer the free energy rapidly increases until reaching its maximum in the middle of the membrane, at approximately 40 Å from the COM, that is, 13.5 and 10.7 kcal mol^–1^ higher than the energy of the preceding minimum for cisPt­(OAc)­(OH) and cisPt­(OAc)_2_, respectively. In correspondence to the interface (**Min_left_**) on the left side of the membrane the interaction of these drugs with the polar head region causes a stabilization of the system by 3.1 kcal mol^–1^ at about 21.5 Å for the cisPt­(OAc)­(OH) and by 1.6 kcal mol^–1^ around the position at 25.4 Å for cisPt­(OAc)_2_ with respect to energy of the drugs located in the bulk solvent. The insertion of the drugs, therefore, in the polar region of the membrane for the efflux toward the extracellular environment, is slightly more favorable.

#### 2.2.2 Interaction of Pt­(IV) Drugs with the Membrane Bilayer. Analysis of Contacts, Hydrogen Bonds, MM-GBSA, and Hydration

##### 2.2.2.1 Analysis of Contacts

The Pt­(IV)-dihydroxido derivative shows a distinctive interaction profile compared with cisPt (Figure S6). Hydroxido groups make the complex even more hydrophilic and polar, which can be seen clearly in its strong hydration shell and more specific interactions with CHL and lipids of the inner region as it crosses the bilayer. In bulk water, cisPt­(OH)_2_ is almost entirely solvated and surrounded by a dense and tightly bound hydration shell. Interactions with lipid heads are minimal, weak, and distant. It is more hydrated than cisPt but less hydrated than cisPt­(OAc)_2_ at this stage. Approaching the interface, cisPt­(OH)_2_ makes 3 native and 65 non-native contacts with OL at 2.3 Å, indicating initial recognition of the upper part of acyl chains. It also forms 13 native and 71 non-native contacts with PC at 2.4 Å, which is stronger than those with cisPt­(OAc)_2_ at this location (Figure S7). Contacts with PE remain weaker, at 3.4 Å, while PG remains distant (6.5 Å). Water continues to dominate, with 1 native and 244 non-native contacts at 1.8 Å, showing that hydration is only partly diminished at the interface. Once in the bilayer core, cisPt­(OH)_2_ displays a different pattern with respect to cisPt, which in order to penetrate deeply necessitates the drag of some water molecules to make the core less lipophilic. CHL contacts imply that the hydroxido ligands may favor insertion into or stabilization of sterol rich regions. At 23 Å from the COM the complex shows a balanced pattern of contacts with water and membrane components, indicating that partial rehydration and return to a more polar environment take place. The dihydroxido derivative penetrates, maintaining significative water and CHL interactions, suggesting that its transport across the membrane might involve transient pores or defects stabilized by sterols rather than pure diffusion through the lipid phase.

The asymmetric cisPt­(OAc)­(OH) complex (Figure S8), bearing one hydroxido and one acetato group in axial positions, exhibits an interaction pattern that lies between those of the two cisPt­(OAc)_2_ and cisPt­(OH)_2_ derivatives, reflecting the balance between a polar hydroxido ligand, which favors the hydration, and a more lipophilic acetate, which enhances the interactions with the hydrophobic region. In the water phase, the complex is strongly hydrated, less than the dihydroxido derivative and more than the diacetato form, while lipid contacts are minimal and distant. At the interface, the complex interacts moderately with CHL and more strongly with OL. Water still dominates, even if hydration starts to weaken in comparison with the bulk. The intermediate behavior of this drug causes a stronger lipid engagement than cisPt­(OH)_2_ but more hydration than the cisPt­(OAc)_2_. Inside the bilayer, it shows 13 native and 510 non-native contacts with OL at 1.9 Å, similarly to the diacetate form, and 79 non-native contacts with CHL at 2.0 Å. Interestingly, nearly all water contacts disappear, showing that the complex has mostly lost its hydration shell. Compared with cisPt­(OH)_2_, which still retains some water molecules and binds strongly CHL in the core, cisPt­(OAc)­(OH) behaves more like the diacetate derivative, penetrating the lipid acyl region and losing water. At the opposite interface, the mixed Pt­(IV) complex rebuilds the hydration shell, while it appears to be significantly hydrated in the aqueous phase. As anticipated, the OAc group facilitates lipid entry, while the hydroxido ligand facilitates hydration and hydrogen bonding formation.

The Pt­(IV) complex bearing two axial acetato groups, cisPt­(OAc)_2_, in the water phase, at 70 Å, is strongly hydrated, and the hydration shell is much more extended and tightly bound than that observed for cisPt, while contacts with lipid groups are almost negligible. At the interface, at 58 Å, cisPt­(OAc)_2_ starts to interact with polar lipid headgroups but in a distinct fashion with respect to cisPt. In fact, the complex appears to be involved in strong and persistent interactions with the lipid carbonyl/oxygen environment. Interactions with PC are weaker if compared to cisPt, which instead tends to prefer PC heads at this position. Concerning water contacts at the interfaces, they decrease, indicating a substantial hydration but less dominant than in the bulk. In the membrane core, at 40 Å, cisPt­(OAc)_2_ native contacts with CHL are not present, but a very large number of non-native contacts are established, also with OL, at 2.0 Å. These data suggest that unlike cisPt, which maintains a relatively balanced interaction between water pockets and lipids, it embeds more directly into the apolar lipid phases, establishing extensive but dynamic interactions with acyl carbonyl/ester regions. The almost complete absence of native water contacts implies that the complex releases most of its hydration shell crossing the hydrophobic barrier, although some water molecules may still be present. At the opposite interface, cisPt­(OAc)_2_ regains some hydration (1 native and 206 non-native water contacts at 1.9 Å) and forms persistent interactions with OL, while CHL shows only sporadic interactions. Therefore, the compound crosses the membrane without binding strongly to CHL, unlike cisPt. A denser and more tightly bound hydration shell surrounds cisPt­(OAc)_2_ in the aqueous phase than cisPt, in agreement with its higher oxidation state and the presence of axial acetates, which reinforces the polarity and hydrogen bond formation capacity. Strong interactions are established with OL tails more than with PC and a deeper but more dynamic partitioning into the bilayer hydrophobic region is observed. In contrast cisPt establishes more balanced interactions with water and PC, suggesting that axial acetates modulate membrane permeability and orientation.

##### 2.2.2.2 Hydrogen Bonds

The three examined Pt­(IV) derivatives, albeit with some differences, show a common behavior that is analogous to that of cisPt. Hydrogen bonds established with water molecules in bulk water moving toward the interface region begin to be substituted by new interactions not only with various lipid heads but also irregularly with the upper portion of lipid tails. Water molecules, however, continue to be the preferred partners for hydrogen bonding. Within the hydrophobic bilayer core, hydrogen bond formation becomes less frequent and short-lived because of the nonpolar nature of the environment, with CHL and OL tails being the favored partners. As confirmed by water hydrogen bonds detection and analogously to cisPt, both cisPt­(OH)_2_ and cisPt­(OAc)­(OH), but not cisPt­(OAc)_2_, drag water molecules into the center of the bilayer, reducing its hydrophobicity. The number of hydrogen bonds with water increases once again when the drugs permeate the membrane, reaching the intracellular environment, maintaining some contacts with CHL or OL, and establishing new hydrogen bonds with polar heads. The whole behavior is consistent with the change of the local polarity of the surrounding environment.

##### 2.2.2.3 MM-GBSA

The values of the single terms of the total MM-GBSA energy at the three key positions, 58 and 23 Å (interfaces) and 40 Å (core), for the translocation process are reported in Table S1 of the Supporting Information. The trend in behavior of the three Pt­(IV) derivatives reproduces that of the cisPt precursor. The small positive values of Δ*G*_total_ of about 1.3 kcal mol^–1^ for the penetration in the bulk water solvent (70 Å) become more favorable, moving toward the two interfaces while they decrease again in correspondence to the bilayer core. The Δ*E*_vdW_ contribution and together the electrostatic contribution, Δ*E*_ele_, are highly stabilizing and represent the driving force of the whole permeation process, becoming substantially more negative as the complex moves into the membrane, especially in correspondence to **Min_right_** and **Min_left_** interface positions. The interactions established by the drugs with charged and polar groups of the membrane constituent lipids drive membrane penetration. In correspondence to the minima, these interactions, as expected, are more favorable, especially at the second interface, when the drugs come in contact with the polar part of the bilayer and the aqueous environment, while their strength significantly decreases at the center of the membrane, where the drug is in contact with the apolar ends of the lipid tails. In all systems, Δ*G*_GB_, the electrostatic component of the solvation energy, has a destabilizing effect. In addition, the solvation effect is unfavorable at minima, where it changes in a range between 22 and 37 kcal mol^–1^, more than in correspondence to the bilayer center, included between 11 and 21 kcal mol^–1^. The Δ*G*_SA_ term, which is the contribution of the solvation energy due the nonpolar part and takes into account the hydrophobic effects, has slightly stabilizing effects for all considered systems, tendentially more in the core than at the interfaces. This effect, in agreement with the polarity of complexes, is more stabilizing for the diacetate than the mixed and dihydroxido derivatives. The negative values of the Δ*G*_gas_ term for all the considered systems indicate that the drugs are already in a favorable stable conformation prior to the interaction with the membrane. The Δ*E*_vdW_ contributions reflect the membrane affinity of the complexes, highlighting the dominant role of hydrophobic contacts for the membrane partitioning that follows the order cisPt­(OAc)_2_ > cisPt­(OAc)­(OH) ≫ cisPt­(OH)_2_ ≈ cisPt. Indeed, it is possible to infer from such analysis that replacing hydroxido ligands with acetato groups increases hydrophobic contacts and strongly enhances membrane association, affecting the uptake and localization in the membrane environment. In conclusion, cisPt­(OAc)_2_ is the strongest binder everywhere inside the membrane, especially due to the Δ*E*_vdW_ contribution due to the presence of acetates that establish hydrophobic contacts. The asymmetric cisPt­(OAc)­(OH) derivative follows in the series, interacting well with the membrane, as proven by Δ*E*_vdW_ contributions compensated by electrostatic penalties larger than those for the diacetate in some positions. cisPt binds membrane components moderately, while cisPt­(OH)_2_ is the weakest binder in the membrane due to the presence of two hydroxido ligands, which increase the polarity and reduce hydrophobic contacts compared to acetato ligands and reduce consequently favorable Δ*E*_vdW_ contributions.

##### 2.2.2.4 Hydration of Pt­(IV) Complexes

The profiles, shown in Figure 2, reporting the number of water molecules surrounding the four Pt complexes as a function of their positions in the membrane, follow the same trend. The number of water molecules gradually decreases going from the bulk solvent to the polar head region whose hydrophilic nature favors the interaction with the water molecules, while the hydrophobic character of the lipid chains reduces the accessibility of the water molecules in this region. The number of molecules in the water shells of cisPt­(OH)_2_, according to the similarity between their PMF profiles, is comparable to that in the water shell of cisPt and starting from 15 molecules in the bulk, such a number decreases during the permeation becoming 5 at the interface. Going toward the interleaflet region this number further decreases although both compounds drag water into the membrane interior and their penetration into the lipophilic environment is assisted by the persistent presence of solvent molecules. On the contrary, the number of molecules accompanying the penetration of the two cisPt­(OAc)­(OH) and cisPt­(OAc)_2_ complexes is larger at the entrance, 17 and 19, respectively, and drastically diminishes proceeding toward the bilayer center, as the more lipophilic nature of the acetato ligands allows them to substitute interactions with water by forming new interactions with the various components of the membrane.

##### 2.2.2.5 Diffusion, Permeability, and Resistance of Pt­(IV) Complexes

The computed D­(*z*) profiles shown in Figure 3 indicate that the diffusion trend of the four considered complexes is opposite to that extracted from PMF profiles: cisPt exhibits the highest local diffusivity across much of the membrane coordinate, followed by cisPt­(OH)_2_, cisPt­(OAc)­(OH), and finally cisPt­(OAc)_2_ having the lowest diffusivity. This trend reflects the hydrodynamic and steric constraints experienced by the complexes. In fact, cisPt and cisPt­(OH)_2_ are small and less sterically hindered, resulting in higher effective mobility when they are already within a given environment, no matter if they are aqueous or lipidic. In addition, in lipid membranes, the disordered hydrocarbon region can enhance the high mobility of small drugs. In contrast, larger complexes, like cisPt­(OAc)­(OH) and cisPt­(OAc)_2_, exhibit reduced local diffusivity due to increased resistance opposed by the lipid environment.

However, as already stated and observed in other studies,^35^ local diffusivity is sensitively less determinant for the overall permeability compared to the free-energy landscape. Further information can be extracted from the graphs of the radial distribution function, *g*(*r*), reported in Figure S4 of the Supporting Information for cisPt and its Pt­(IV) derivatives in water at the three key positions, 58 and 23 Å (interfaces) and 40 Å (core), of the PMF profile.

## 3 Conclusions

In the effort of improving the cytotoxic efficacy of Pt-based anticancer compounds, octahedral Pt­(IV) complexes, being significantly more inert, offer several advantages over their Pt­(II) precursors in terms of lower reactivity and probability to arrive at the tumor site intact by avoiding side reactions with biomolecules due to their greater kinetic inertness. Despite their undoubted advantages, many Pt­(IV) complexes have been admitted to clinical trials, but none of them have been approved yet because of their inferior overall performance in clinical practice compared to existing treatments. As cellular uptake and accumulation are critical steps in the whole mechanism of action, allowing us to discriminate drugs that can result in being efficacious, in the present paper a computational investigation of the permeation process of cisplatin and its three Pt­(IV) derivatives through a realistic plasma membrane prototype of human breast cancer cells has been undertaken by means of biased MD. Calculated PMF profiles highlight that the main obstacle to permeation is represented by the barrier calculated in correspondence of the center of the membrane. However, this barrier in the lipophilic tail region, calculated with respect to the previous minimum, is higher for cisPt (16.8 kcal mol^–1^) and cisPt­(OH)_2_ (15.8 kcal mol^–1^) than cisPt­(OAc)­(OH) (13.5 kcal mol^–1^) and cisPt­(OAc)_2_ (10.5 kcal mol^–1^). Likewise, the free energy minima in which cisPt (−0.8 kcal mol^–1^) and cisPt­(OH)_2_ (−0.5 kcal mol^–1^) are weakly trapped when the drugs pass from bulk water to the polar headgroup region are less deep than the free energy minima of −2.7 and −1.3 kcal mol^–1^, characterizing the permeation profiles of cisPt­(OAc)­(OH) and cisPt­(OAc)_2_. The factor that governs the most significant differences in behavior is the presence of the more lipophilic acetato ligand. Indeed, while the hydroxido ligand facilitates hydration and hydrogen bonding formation, the acetate is able to both interact with the polar region of the membrane and to favor the interactions with the hydrophobic region. Additionally, cisPt and the symmetric cisPt­(OH)_2_ are able to drag water molecules into the center of the bilayer, as hydration profiles show, to assist them in penetrating in the hydrophobic core. cisPt­(OAc)­(OH) and cisPt­(OAc)_2_, instead, are strongly hydrated in the extracellular medium, while the contacts with the water molecules are substituted by the interaction with the membrane components during the permeation and almost completely lost in the center of the bilayer. This comparison between the permeation process of cisPt and its three Pt­(IV) derivatives aimed at exploring differences in behavior that could represent additional factors leading to cytotoxic profiles for Pt­(IV) compounds that are noncompetitive with those of the FDA-approved parent Pt­(II) complexes despite their many advantageous features. The outcomes of the present biased MD simulation, the first exploring the passive diffusion of Pt­(IV) products, demonstrate that the identity of the axial ligands, as it has been assumed, has an influence on the passive diffusion through the plasma membrane, and specifically, the presence of acetato ligands facilitates penetration and accumulation. Additionally, this study proves that passive diffusion is the common permeation mechanism to the three complexes, and eventually, only for the cisPt­(OAc)­(OH) complex should an additional and reinforcing assisted transportation mechanism be operative that justifies its superior cytotoxic activity detected experimentally. Systematic studies of the whole mechanism of action, starting from the cellular uptake first step, of, in principle, very efficacious Pt­(IV) prodrugs are underway with the purpose of accumulating information that is helpful for overcoming the gap between their assumed efficacy and clinical failure.

## 4 Methods

### 4.1 Molecular Models and Force Fields Parametrization

For platinum complexes investigated in this work, cisPt, cisPt­(OH)_2_, cisPt­(OAc)­(OH), and cisPt­(OAc)_2_ shown in Scheme 1, molecular-mechanics force field parameters have been obtained by using easyPARM v2.00,^36^ a Python-based tool, specifically designed to automatize the parametrization of transition-metal complexes, by combining the Seminario method^37^ and the AMBER^38^ and GAFF^39^ libraries. The xyz coordinates, the Cartesian Hessian matrix to extract the vibrational force constants, and the atomic charges of the complexes have been obtained by calculations with the Gaussian 16 ^40^ QM code at the default numerical precision. All metal complexes have been optimized within the density functional theory (DFT) framework employing the hybrid and generalized gradient approximation (GGA) B3LYP^41–43^ functional in combination with the 6–31G* basis set for all atoms except the metal, described by the LANL2DZ relativistic pseudopotential and valence basis set.^44^ During geometry optimization, *C*_2V_ symmetry, except for the cisPt­(OAc)­(OH) complex, has been imposed. Following optimization, frequency calculations within the normal mode approximation have been performed at the same level of theory to compute the Cartesian Hessian matrixes, confirming the absence of any negative eigenvalue of Hessian. Atomic charges have been provided through the restricted electrostatic potential (ESP)^45,46^ method using the same protocol, while LJ parameters have been retrieved from UFF.^47^

The same model used by Almeida and co-workers has been adopted as the membrane model for the evaluation of platinum drugs’ cellular uptake^27^ to mimic plasma membranes of breast cancer. This double-membrane model, shown in Figure 4 composed by the main lipids found in plasma membranes of breast cancer cells,^48^ considers the loss of asymmetric nature among the two leaflets and other molecular biomarkers proved to be present in breast cancer cells, such as the phosphatidylserine (PS) expression at the outer leaflet, and an enriched concentration of phosphatidylethanolamine (PE) and cholesterol (CHL) in comparison to normal cells.^49^ Phospho-rac-glycerol (PG) and phosphocholine (PC) lipids in the membrane model are also present. The acyl chains of breast cancer cell membranes are mainly monounsaturated fatty acids 18:1;^50^ they included dioleoyl chains (DO) in all phospholipids of the membrane model. The structures of the cited lipids are depicted in Figure S9 of the Supporting Information. The two double layers have the same lipid composition. The plasma membrane model has been described by the parameters of the Lipid21 Amber force field.^51^ A peculiarity of this model, built as double membrane, is the asymmetric concentration of the main ions, Na^+^, K^+^, and Mg^2+^, to mimic the cytoplasm and the extracellular regions of a breast cancer cell.^52–54^ To neutralize the system, Cl^–^ ions have been added, and their final distribution reproduced in part the concentration gradient detected in cells. Water molecules have been described with the TIP3P model,^55^ while the monovalent ions Na^+^, K^+^, and Cl^–^ with parameters developed by Joung et al.,^56^ and the bivalent Mg^2+^ by using parameters of Li and Kenneth.^57^

**Figure 4:**
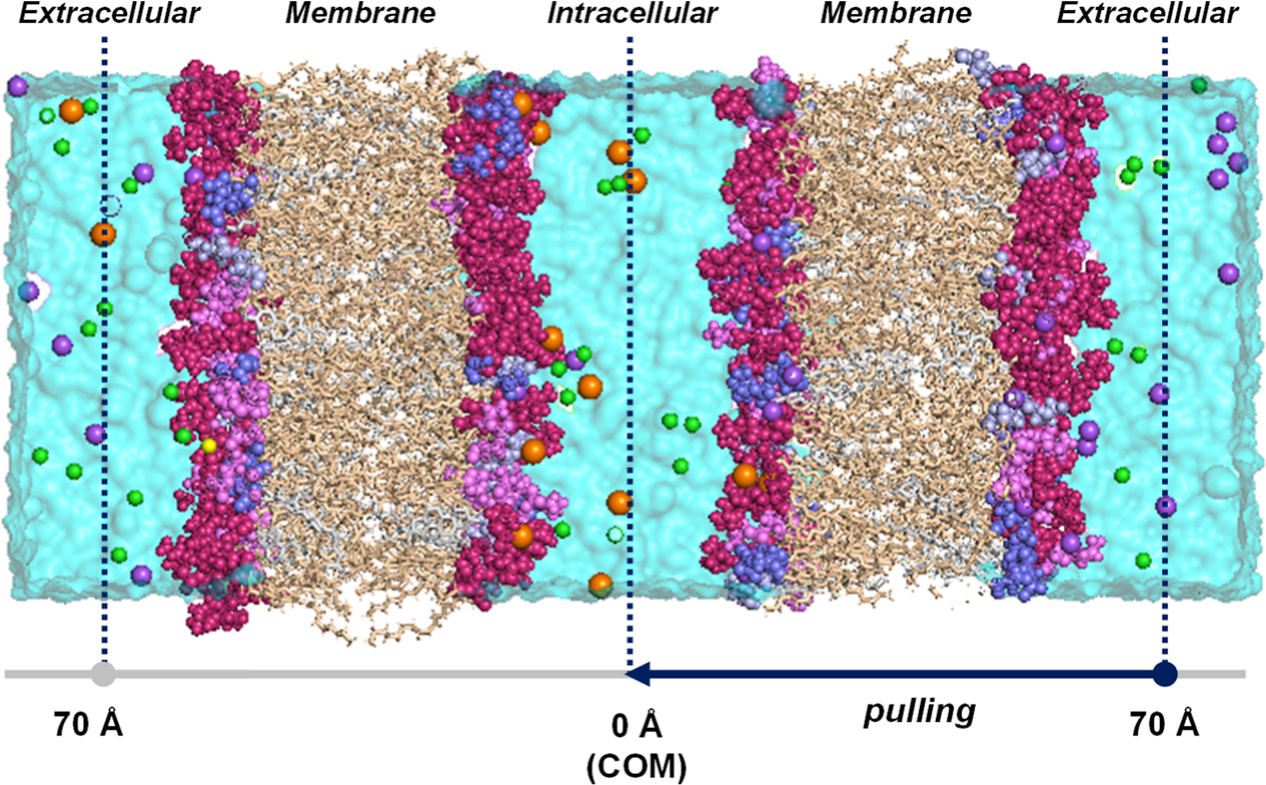
Membrane model of a human breast cancer cell. System components and their color code: water (cyan), PC heads (warm pink), PE heads (violet), PS heads (slate), PG heads (light blue), OL tails (wheat), CHL (gray), K^+^ (orange), Na^+^ (purple), Mg^2+^ (yellow), Cl^–^ (green).

### 4.2 MD Simulations Details

The MD simulations have been carried out with the Amber22 code^38^ employing the pmemd CUDA implementation.^58–60^ The equilibration of the membrane system began with a two-step minimization process. In the first stage the solvent has been minimized with 40000 steps restraining the membrane with a force constant of 500 kcal mol^–1^ Å^–2^. The entire system without applying any positional restraints has been involved in the second minimization of 9000 steps. Both stages combined the steepest descent (SD) algorithm in the initial half with the conjugate gradient (CG) approach in the latter one.^61^ Once the minimization has been completed, the system has undergone a heating phase, gradually raising the temperature from 283 to 310 K under the *NVT* ensemble for 20 ns and applying a positional restraint of 10 kcal mol^–1^ Å^–2^ on the membrane. During this step, temperature control has been ensured with a Langevin thermostat operating at a collision frequency of 1.0 ps^–1^.^62^ The subsequent equilibration has been conducted in the *NPT* ensemble for 30 ns, using the Langevin thermostat in combination with the anisotropic Berendsen barostat,^63^ maintaining a reference pressure of 1.0 bar with a relaxation time of 1.0 ps. Finally, a production molecular dynamics simulation has been conducted for 200 ns, at 310 K, in the NTP ensemble, with periodic boundary conditions, employing the Langevin thermostat with a damping coefficient of 1 ps^–1^ and the Berendsen barostat to maintain isotropic pressure. A time step of 2 fs has been used, with all covalent bonds involving hydrogen atoms constrained using the SHAKE algorithm. Long-range electrostatic interactions have been treated using the particle-mesh Ewald method, with a cutoff of 10 Å. The final snapshot of the production phase has been adopted as the starting configuration of the simulations involving metallodrugs.

To evaluate the influx processes, each platinum complex has been initially placed at 70 Å away from the membrane model center of mass (COM), within the aqueous extracellular-mimicking environment (Figure 4). The translocation of the platinum drugs across the membrane has been studied by combining steered molecular dynamics (SMD)^64^ with umbrella sampling (US)^65^ techniques.

Before the drug-pulling step, each system has been minimized to relax further water and ions and to eliminate unfavorable contacts between the solvent and the introduced permeant, and a short thermalization (5 ns) at 310 K and at constant volume, similar to the protocol already adopted for the membrane alone. Then, each system has been equilibrated for 20 ns, with a harmonic restraint of 2.5 kcal mol^–1^ applied to the drug at 70 Å. During SMD the COM of each drug has been pulled from 70 Å toward the center of the membrane, cytoplasm-like region, along the axis parallel to the membrane normal, for 72 ns, at a constant velocity of 0.972 Å ns^–1^, within the NPγT ensemble regulated by the semi-isotropic Berendsen barostat and controlled surface tension (γ). From these SMD trajectories, 71 frames, each separated by 1 Å from the next one, have been extracted to serve as the starting points for the US windows. Every window has been equilibrated first for 20 ns, followed by a 60 ns production run in the NPγT ensemble, applying a harmonic biasing potential of 2.5 kcal mol^–1^ Å^–2^. For the potential of mean force (PMF) calculations, only the final 30 ns of each production simulation has been considered, providing a cumulative sampling time of 2.13 μs. To reconstruct PMFs, the Weighted Histogram Analysis Method (WHAM)^66–69^ has been employed, while statistical uncertainties have been estimated using bootstrap error analysis.^67,70^ In addition, to maintain bond stability, all bonds involving hydrogen atoms have been constrained with the SHAKE algorithm.^71^ Throughout the simulations, a 2 fs time step and periodic boundary conditions have been applied. For each system, analysis of contacts, hydrogen bonds, hydration, radial distribution function, electron density profiles for various groups, and the Molecular Mechanics-Generalized Born (MM-GBSA) have been computed considering only the last 30 ns of the windows production and using CPPTRAJ.^72^

The MM-GBSA (Molecular Mechanics-Generalized Born Surface Area), developed by Kollman and co-workers,^73–75^ is a computational method used to estimate the free energy between two or more interacting systems. Here, it has been adopted to study the affinity between the membrane model and the investigated platinum complexes, cisPt and its Pt­(IV) derivatives. The method combines Molecular Mechanics (MM) and the Generalized Born and Surface Area solvation models (GBSA). The MM part calculates the internal energy, the Generalized Born (GB) model approximates the solvation energy, and the Surface Area (SA) the solvation energy. Estimating the binding free energy makes it possible to characterize the interaction strength and the affinity between the drug and the biological system under consideration. It consists of bonding terms (stretching, angle bending, torsional energy) and non-bonding terms including electrostatic and van der Waals interactions. Each term is calculated for both the complexes and the individual isolated components, from which the total energy of the system is obtained as

$${\Delta G}_{total}=G_{membrane-complex}-G_{membrane}-G_{complex}$$

The expression described above can also be defined as the sum of several contributions

$${\Delta G}_{total}=\Delta H-T\Delta S={\Delta E}_{MM}+{\Delta G}_{sol}-T\Delta S$$

$${\Delta E}_{MM}={\Delta E}_{\mathrm{int}}+{\Delta E}_{ele}+{\Delta E}_{vdW}$$

$${\Delta G}_{sol}={\Delta G}_{GB}+{\Delta G}_{SA}$$

Δ*E*_MM_ is related to the gas-phase molecular mechanics (MM) variations, considering changes in internal energy (Δ*E*_int_), electrostatic energy (Δ*E*_ele_) and van der Waals energy (Δ*E*_vdW_). Δ*G*_sol_ is the solvation energy, which can be decomposed into a polar contribution (Δ*G*_GB_) concerning electrostatic solvation energy, and a nonpolar contribution (Δ*G*_SA_) between the solute and the continuous solvent. Δ*G*_GB_ is calculated by using the GB method, and Δ*G*_SA_ by using the solvent-accessible surface area.^76,77^ The term -*T*Δ*S* is the change in the conformational entropy. However, this term due to the high computational cost and the low prediction accuracy is often omitted.^78–84^ The accuracy of the free energy values computed with the MM-GBSA method may be affected by multiple errors, such as the length of the MD simulation considered being system-dependent, the presence of various substates, the charge model,^85^ the solvation method,^86^ the sampling,^87^ the force field,^85^ dielectric constant,^88^ and the entropy associated with conformational variations.^89^ Furthermore, the discrepancy of the values obtained with the MM-GBSA, if compared to those calculated with the US (WHAM), is mainly attributable to the neglect of the entropic contribution due to conformational changes,^78,83^ and particularly relevant in membrane systems due to lipid fluctuations.

The permeability (P) of the platinum compounds has been estimated by applying the inhomogeneous solubility-diffusion theory,^34^ formalized in eq 1

1 $$P={\left(\int_{z_{in}}^{z_{out}}\frac{e^{\beta G\left(z\right)}}{D\left(z\right)}dz\right)}^{-1}$$

In this equation, β is defined as 1/*k*_B_ *T* (*k*_B_ is the Boltzmann constant and *T* is the absolute temperature), while D­(*z*) and G­(*z*) are the local diffusion coefficient and the free-energy profile obtained from the potential of mean force of the drug at a given position z along the membrane normal, respectively. D­(*z*) has been evaluated following the approach proposed by Hummer,^90^ which relies on both the positional variance along the *z* direction and the time autocorrelation function C_*zz*_ of the *z* coordinate, as described in eq 2

2 $$D\left(z\right)=\frac{{var\left(z\right)}^{2}}{\int_{0}^{\infty }C_{zz}\left(t\right)dt}$$

The autocorrelation function *C*_*zz*_(*t*)^91^ contained in eq 2 has been computed as reported in eq 3

3 $$C_{zz}\left(t\right)=\frac{1}{n_{samples}}\sum_{i=0}^{n_{samples}-1}\delta_{z}\left(i\right)\delta_{z}\left(t+i\right)$$

considering 195000 samples and the positional fluctuation d_*z*_(*t*).

The overall membrane resistance *R*_eff_, the reciprocal of *P*, can also be obtained by integrating the local resistance *R*(*z*), as reported in eq 4

4 $$\frac{1}{P}=R_{eff}=\int_{z_{in}}^{z_{out}}R\left(z\right)dz$$

## Supplementary Material



## Data Availability

All data generated or analyzed during this study are included in this published article and its Supporting Information file.

## Notes

Histogram plots of the sampled distributions obtained for the US windows along the reaction coordinate, the plots of the electron density for various groups and the lipid structures, contact analysis and corresponding heatmap, the values of the single terms of the total energy (*E*_tot_) estimated through MM-GBSA, the radial distribution function for the considered complexes in water, diffusion values, resistance profiles, and chemical structure of the lipids constituting the membrane model (PDF)
