# Supplementary material for: Molecular Dynamics Simulation of Passive Diffusion across a Human Breast Cancer Cell Membrane Model. Comparison between Cisplatin and Its Pt(IV) Derivatives
Source: J Chem Inf Model. 2026 Mar 6;66(6):3150–63. doi: 10.1021/acs.jcim.5c02819 (PMC13014451; doi:10.1021/acs.jcim.5c02819)
Supplement: Supplementary file 1 [file ci5c02819_si_001.pdf]

Supporting Information

Molecular Dynamics simulation of passive diffusion across a human breast cancer cell membrane model. Comparison between cisplatin and its Pt(IV) derivatives

Daniele Belletto[1], Stefano Scoditti[1], Stefano Borocci[2], Nico Sanna[2], Costantino Zazza[2], Emilia Sicilia[1*]

[1]Department of Chemistry and Chemical Technologies, Università della Calabria, Ponte P. Bucci, 87036 Arcavacata di Rende (CS) (Italy).
[2]Department for Innovation in Biology Agro-Food and Forest Systems (DIBAF), University of Tuscia, Largo dell'Università snc, 01100 Viterbo, Italy
emilia.sicilia@unical.it

Figure S1. Distribution histogram plots for the umbrella sampling windows along the reaction coordinate adopted to build the PMF profiles for all the considered systems. A brief histograms' description is also given. S3

Figure S2. Electron Density (ED) profiles for the various groups considered for each platinum complex-membrane system at the interfaces (58 and 23 Å from COM) and at the bilayer center (40 Å from COM). S4

Figure S3. Hystogram plots of the contact analysis and the corresponding heatmap of the average distance between cisPt and the various groups at the four key positions, 70 Å (bulk), 58 and 23 Å (interfaces) and 40 Å (core). S5

Table S1. Values of the single terms of the total energy and their corresponding errors, estimated through MM-GBSA, for all the four investigated complexes in correspondence of interfaces (58.0 and 23.0 Å) and core (40.0 Å) positions. S6

Figure S4. Radial distribution function, *g(r)*, for cisPt and its Pt(IV) derivatives in water at the three key positions, 58 and 23 Å (interfaces) and 40 Å (core), of the PMF profile. S7

Table S2. Diffusion values D(z), expressed in $cm^2 s^{-1}$, extracted from the diffusion profiles for the four complexes. Positions, expressed in Å, at the interfaces ($\mathbf{Min_{right}}$ and $\mathbf{Min_{left}}$) at the membrane bilayer core (**Max**) and in bulk water of extracellular and intracellular medium ($\mathbf{E_m}$ and $\mathbf{I_m}$, respectively) reported in brackets with respect to the COM. S8

Figure S5. Resistance profiles of A) cisPt, B) $cisPt(OH)_2$, C) cisPt(OAc)(OH) and D) $cisPt(OAc)_2$, along the permeation path from the extracellular to the intracellular environment. The dashed vertical grey lines indicate the position of the lipid headgroups. S8

Figure S6. Hystogram plots of the contact analysis and the corresponding heatmap of the average distance between cisPt(OH)$_2$ and the various groups at the four key positions, 70 Å (bulk), 58 and 23 Å (interfaces) and 40 Å (core). S9

Figure S7. Hystogram plots of the contact analysis and the corresponding heatmap of the average distance between cisPt(OAc)$_2$ and the various groups at the four key positions, 70 Å (bulk), 58 and 23 Å (interfaces) and 40 Å (core). S10

Figure S8. Hystogram plots of the contact analysis and the corresponding heatmap of the average distance between cisPt(OAc)(OH) and the various groups at the four key positions, 70 Å (bulk), 58 and 23 Å (interfaces) and 40 Å (core). S11

Figure S9. Chemical structure of the lipids that constitute the membrane model used in this work. S12

References S13

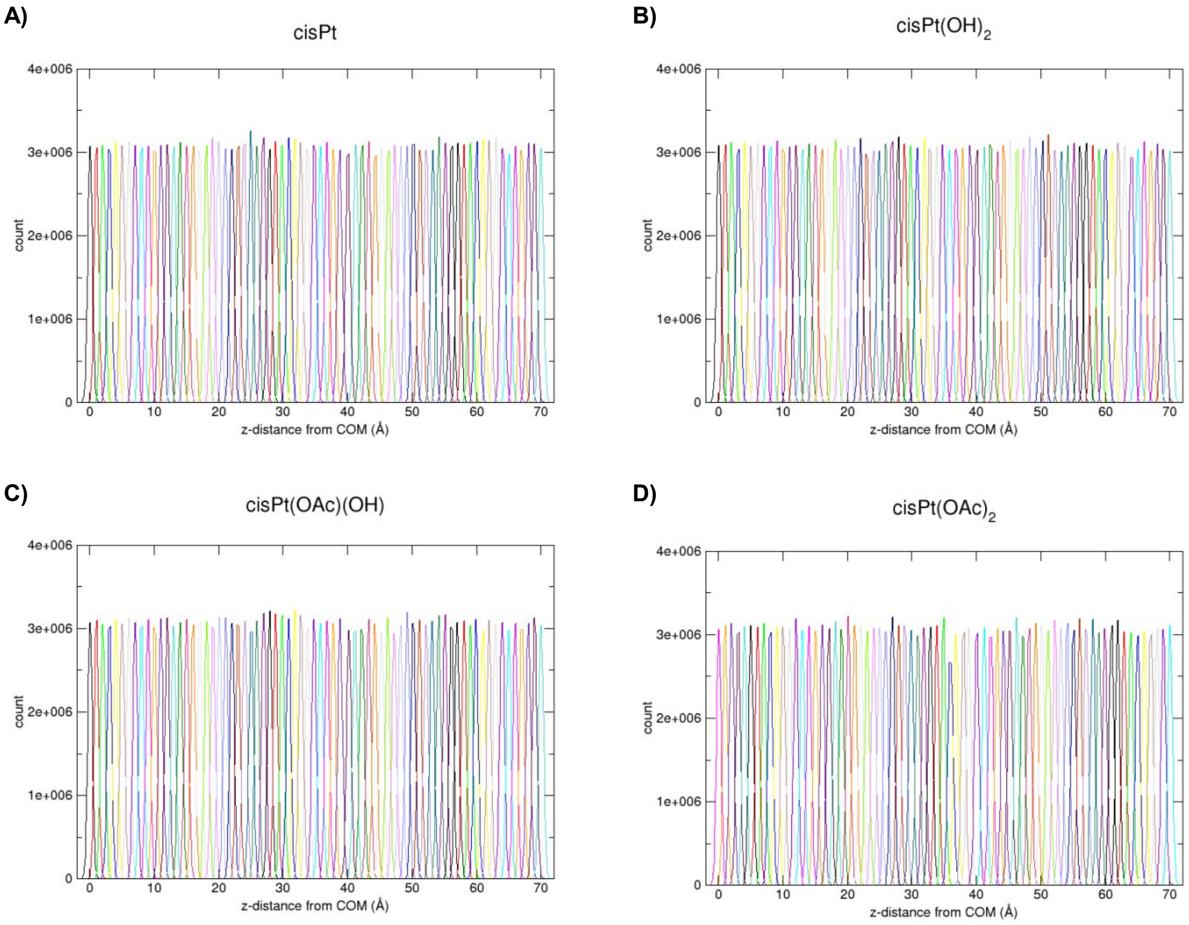

Figure S1. Distribution histogram plots for the umbrella sampling windows along the reaction coordinate adopted to build the PMF profiles for all the considered systems.

The reliability of the sampling and the robustness of the PMF profiles are supported by the histogram plots of the sampled distributions obtained for each umbrella sampling window along the reaction coordinate used to build the energy profiles. In all the cases analyzed, the sampling spans the entire reaction coordinateconsidered in this study, extending from 70 Å to the COM of the full membrane model. Every window effectively covers a portion of the reaction coordinate without evident gaps, ensuring that the WHAM recostruction interpolates the profiles with high accuracy. The sampled distributions are narrow and display consistent and well centered peaks around the restrained position (spaced every 1 Å). They also exhibit regular overlaps of about 30% or more with adiacent windows, an overlap considered adequate for PMF convergence[1–3]. The distributions show comparable amplitudes and areas, indicating that no single window is either under- or over-sampled, thereby minimizing weighting imbalances in the final PMF reconstruction. No discontinuities, outliers, or irregular patterns, such as flattened or bimodal distributions, were detected, confirming good local convergence and absence of issues related to biasing. Moreover, each window contains an high density of sampling points, ensuring extensive data collection that enhances statistical reliabbility and reduces correlation effects. The dense coverage, the uniform distributions, and the consistent overlap between windows enable the accurate identification of energy barriers and local minima with more confidence.

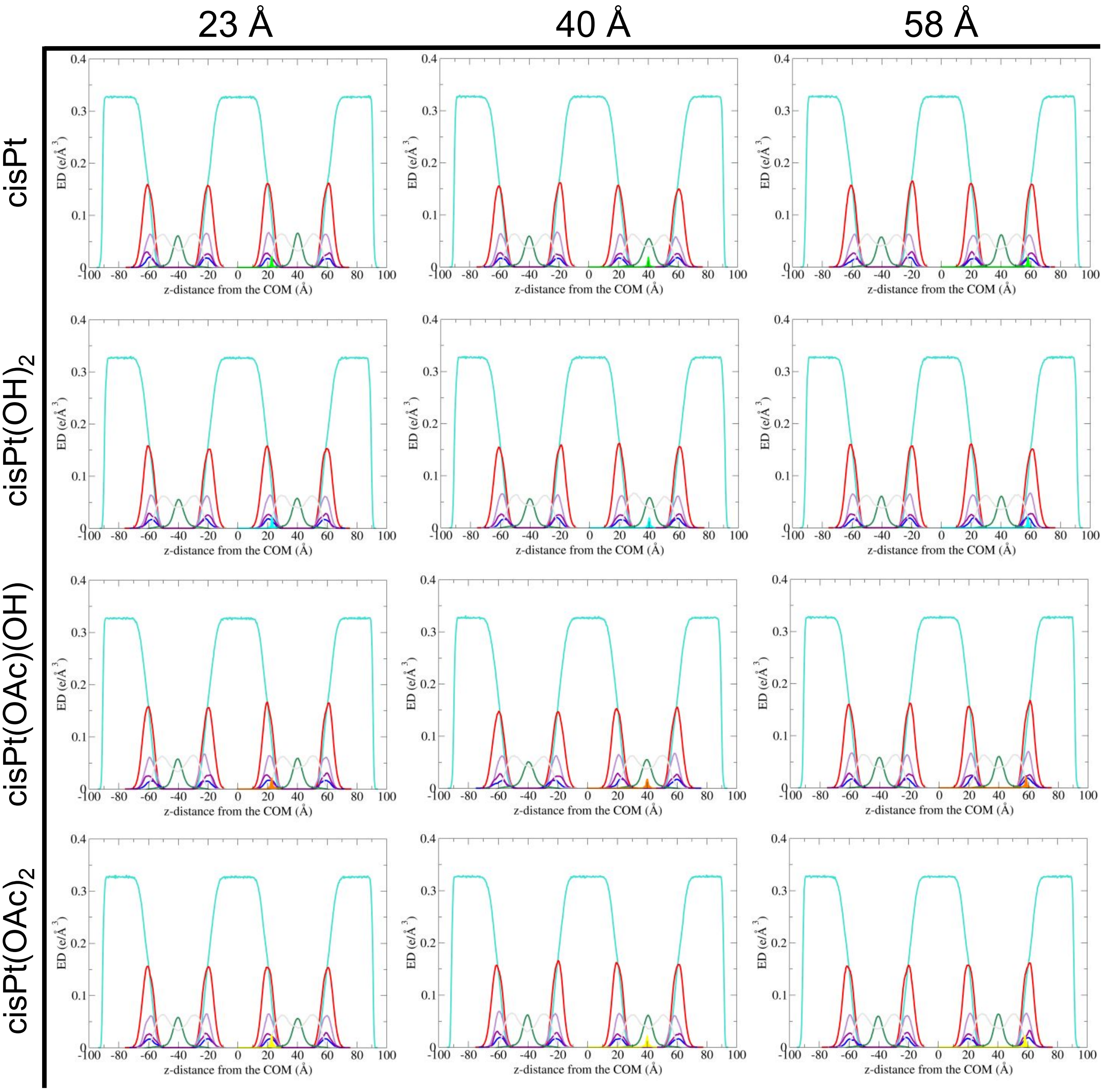

Figure S2. Electron Density (ED) profiles for the various groups considered for each platinum complex-membrane system at the interfaces (58 and 23 Å from COM) and at the bilayer center (40 Å from COM). Color code: cisPt (light green —), cisPt(OH)$_2$ (cyan —), cisPt(OAc)(OH) (orange —), cisPt(OH)$_2$ (yellow —), water (turquoise —), PC heads (red —), PE heads (lilac —), PG heads (blue —), PS heads (purple —), CHL (gray —), and terminal methyl tail groups (dark green —).

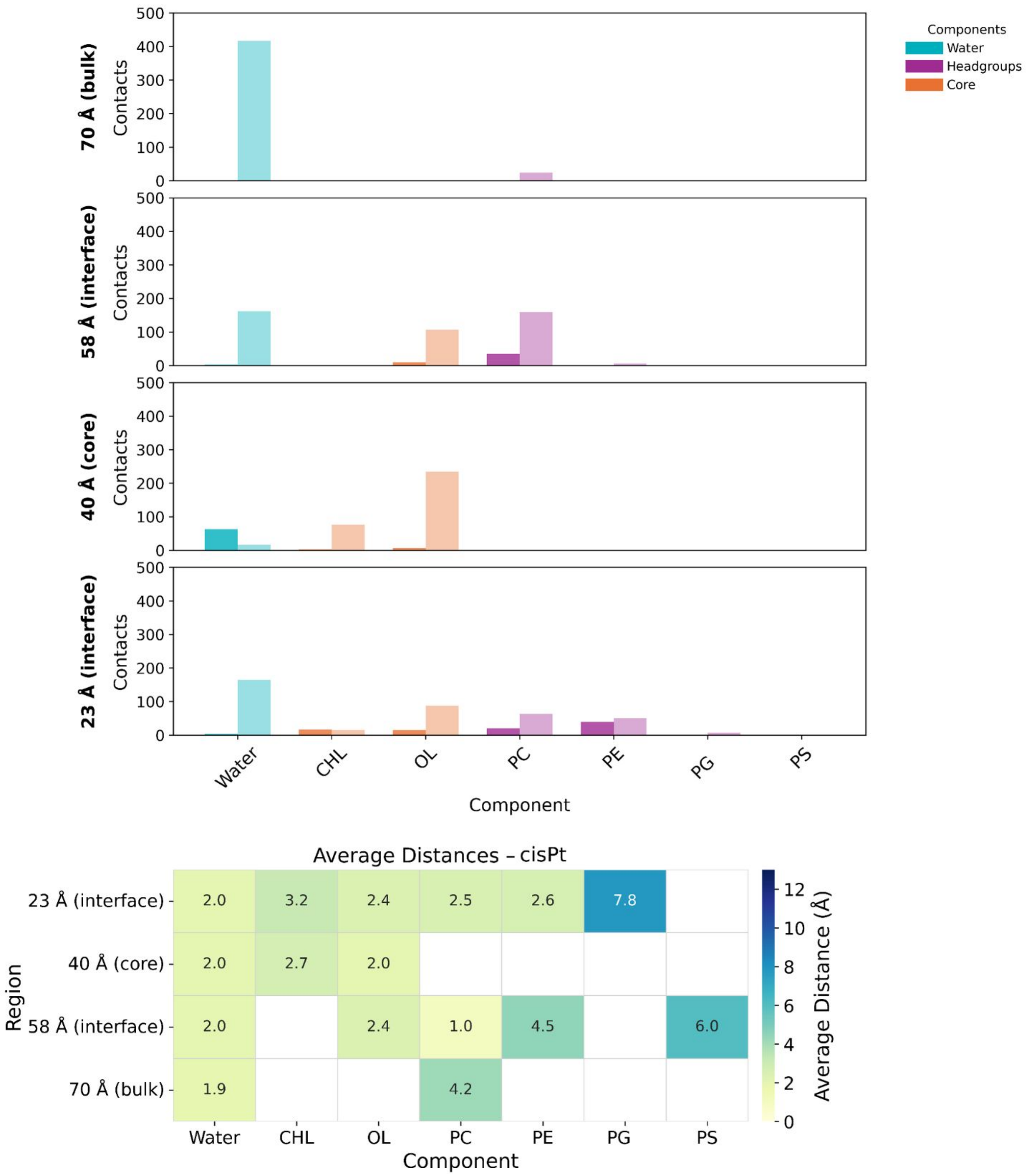

Figure S3. Histogram plots of the contact analysis and the corresponding heatmap of the average distance between cisPt and the various groups at the four key positions, 70 Å (bulk), 58 and 23 Å (interfaces) and 40 Å (core).

Table S1. Values of the single terms of the total energy and their corresponding errors, estimated through MM-GBSA, for all the four investigated complexes in correspondence of interfaces (58.0 and 23.0 Å) and core (40.0 Å) positions.

		58.0	40.0	23.0
E_{VdW}	cisPt	-11.8 ± 2.0	-10.2 ± 1.2	-11.2 ± 2.0
	cisPt(OAc)$_2$	-20.6 ± 2.9	-24.4 ± 2.0	-24.9 ± 2.9
	cisPt(OH)$_2$	-12.1 ± 1.9	-13.4 ± 1.8	-12.5 ± 1.9
	cisPt(OAc)(OH)	-16.3 ± 2.3	-19.6 ± 1.8	-18.2 ± 2.5
E_{elec}	cisPt	-27.1 ± 13.6	-1.2 ± 2.4	-32.5 ± 15.2
	cisPt(OAc)$_2$	-15.4 ± 12.1	-1.3 ± 1.9	-28.9 ± 11.7
	cisPt(OH)$_2$	-22.5 ± 15.2	-11.5 ± 4.2	-18.7 ± 10.2
	cisPt(OAc)(OH)	-12.6 ± 11.0	-3.4 ± 3.4	-24.7 ± 13.7
E_{GB}	cisPt	33.3 ± 11.9	11.5 ± 2.8	35.5 ± 12.4
	cisPt(OAc)$_2$	26.0 ± 9.6	15.1 ± 2.3	36.4 ± 8.1
	cisPt(OH)$_2$	28.8 ± 11.4	20.8 ± 2.6	29.0 ± 7.7
	cisPt(OAc)(OH)	22.4 ± 9.2	13.7 ± 3.9	31.8 ± 10.3
E_{surf}	cisPt	-2.6 ± 0.3	-2.2 ± 0.2	-2.5 ± 0.3
	cisPt(OAc)$_2$	-3.6 ± 0.5	-4.5 ± 0.3	-4.3 ± 0.4
	cisPt(OH)$_2$	-2.7 ± 0.4	-3.2 ± 0.2	-2.6 ± 0.3
	cisPt(OAc)(OH)	-3.1 ± 0.4	-3.9 ± 0.3	-3.5 ± 0.4
ΔG_{gas}	cisPt	-38.9 ± 14.2	-11.4 ± 2.7	-43.7 ± 15.2
	cisPt(OAC)$_2$	-36.0 ± 12.8	-25.7 ± 2.9	-53.8 ± 12.5
	cisPt(OH)$_2$	-34.6 ± 14.2	-24.8 ± 4.0	-31.2 ± 10.5
	cisPt(OAc)(OH)	-28.9 ± 11.7	-23.0 ± 4.0	-42.9 ± 14.6
ΔG_{solv}	cisPt	30.6 ± 11.7	9.3 ± 2.8	33.0 ± 12.3
	cisPt(OAC)$_2$	22.4 ± 9.4	10.6 ± 2.2	32.0 ± 8.0
	cisPt(OH)$_2$	26.1± 11.2	17.6 ± 2.5	26.4 ± 7.5
	cisPt(OAc)(OH)	19.4 ± 9.0	9.8 ± 4.0	28.2 ± 10.1
ΔG_{total}	cisPt	-8.3 ± 4.5	-2.1 ± 2.3	-10.8 ± 5.4
	cisPt(OAc)$_2$	-13.6 ± 4.9	-15.1 ± 3.2	-21.8 ± 6.3
	cisPt(OH)$_2$	-8.5 ± 5.1	-7.2 ± 2.8	-4.8 ± 3.9
	cisPt(OAc)(OH)	-9.6 ± 4.3	-13.2 ± 3.2	-14.7 ± 5.9

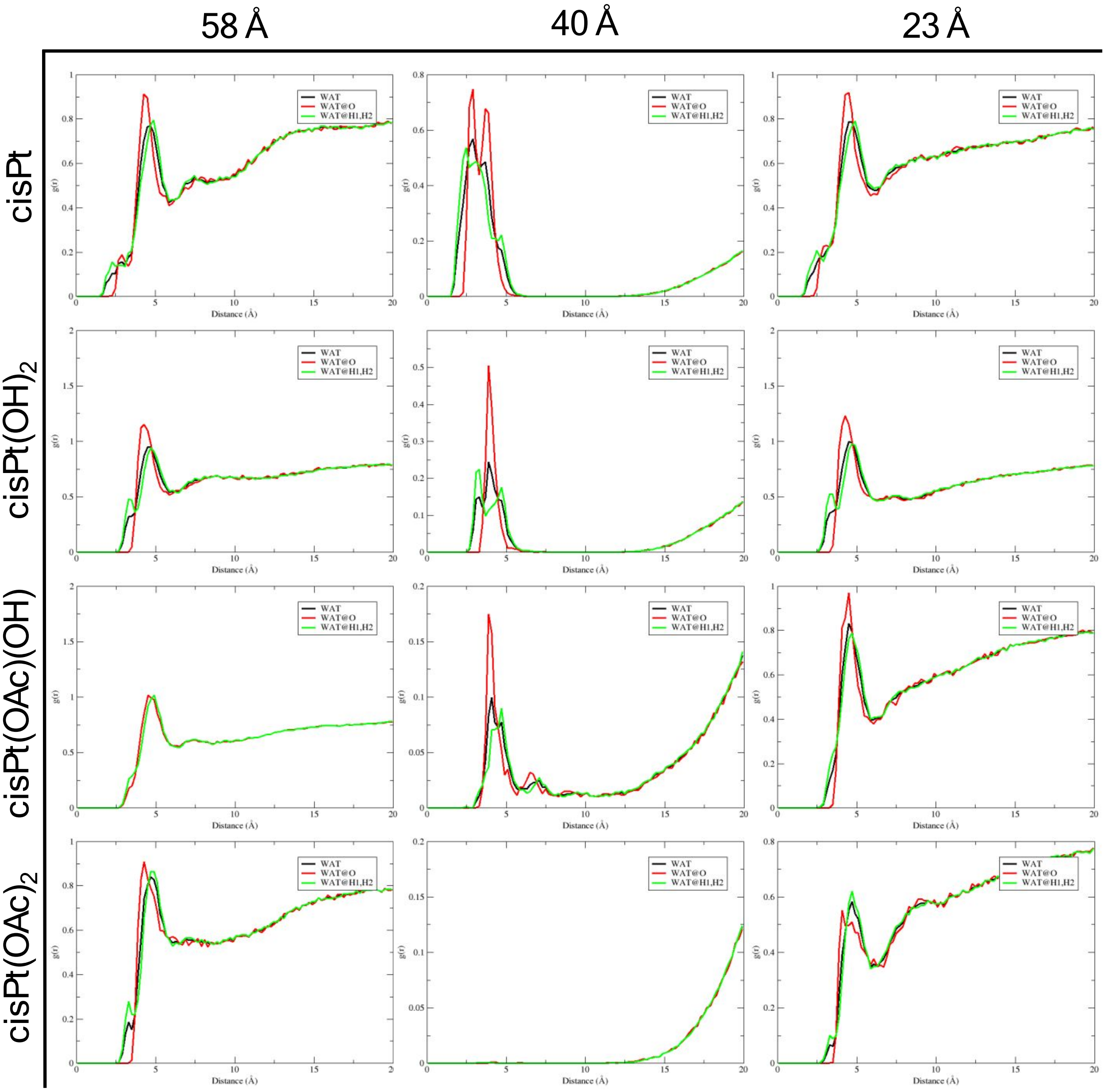

Figure S4. Radial distribution function, *g(r)*, for cisPt and its Pt(IV) derivatives in water at the three key positions, 58 and 23 Å (interfaces) and 40 Å (core), of the PMF profile.

Table S2. Diffusion values D(z), expressed in $cm^2\ s^{-1}$, extracted from the diffusion profiles for the four complexes. Positions, expressed in Å, at the interfaces (**Min_{right}** and **Min_{left}**) at the membrane bilayer core (**Max**) and in bulk water of extracellular and intracellular medium (**E_m** and **I_m**, respectively) reported in brackets with respect to the COM.

	E_m	Min_{right}	Max	Min_{left}	I_m
cisPt	1.3×10^{-5} (70.0 Å)	3.8×10^{-6} (57.3 Å)	7.4×10^{-6} (40.0 Å)	3.6×10^{-6} (22.4 Å)	1.4×10^{-5} (10.0 Å)
cisPt(OH)$_2$	9.4×10^{-6} (70.0 Å)	4.2×10^{-6} (59.4 Å)	4.8×10^{-6} (39.5 Å)	4.5×10^{-6} (23.0 Å)	1.2×10^{-5} (10.0 Å)
cisPt(OAc)(OH)	9.3×10^{-6} (70.0 Å)	4.1×10^{-6} (58.6 Å)	4.7×10^{-6} (40.4 Å)	3.8×10^{-6} (21.5 Å)	1.0×10^{-5} (10.0 Å)
cisPt(OAc)$_2$	8.2×10^{-6} (70.0 Å)	4.3×10^{-6} (58.2 Å)	4.7×10^{-6} (40.5 Å)	3.7×10^{-6} (25.4 Å)	7.5×10^{-6} (10.0 Å)

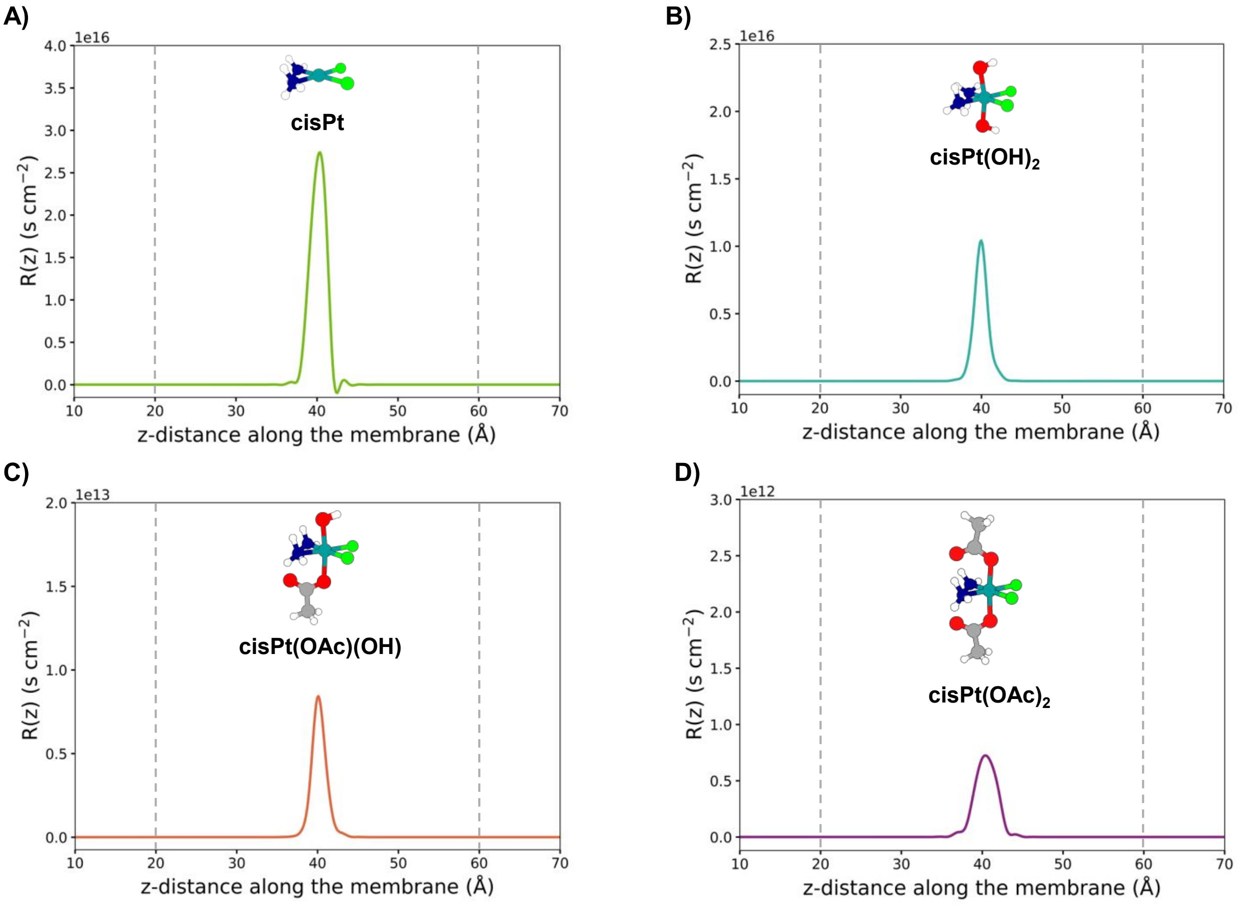

Figure S5. Resistance profiles of A) cisPt, B) cisPt(OH)$_2$, C) cisPt(OAc)(OH) and D) cisPt(OAc)$_2$, along the permeation path from the extracellular to the intracellular environment. The dashed vertical grey lines indicate the position of the lipid headgroups.

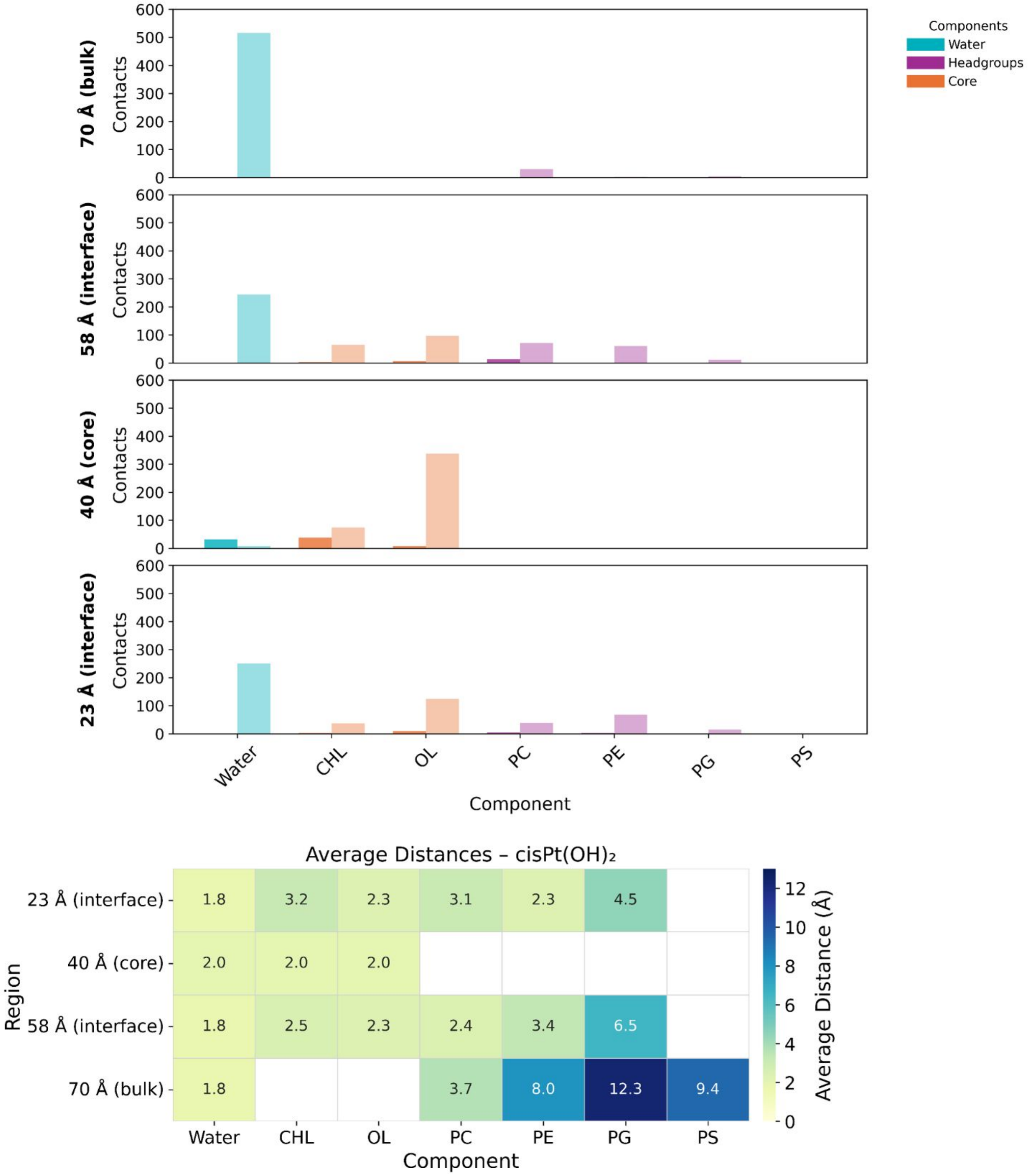

Figure S6. Hystogram plots of the contact analysis and the corresponding heatmap of the average distance between cisPt$(OH)_2$ and the various groups at the four key positions, 70 Å (bulk), 58 and 23 Å (interfaces) and 40 Å (core).

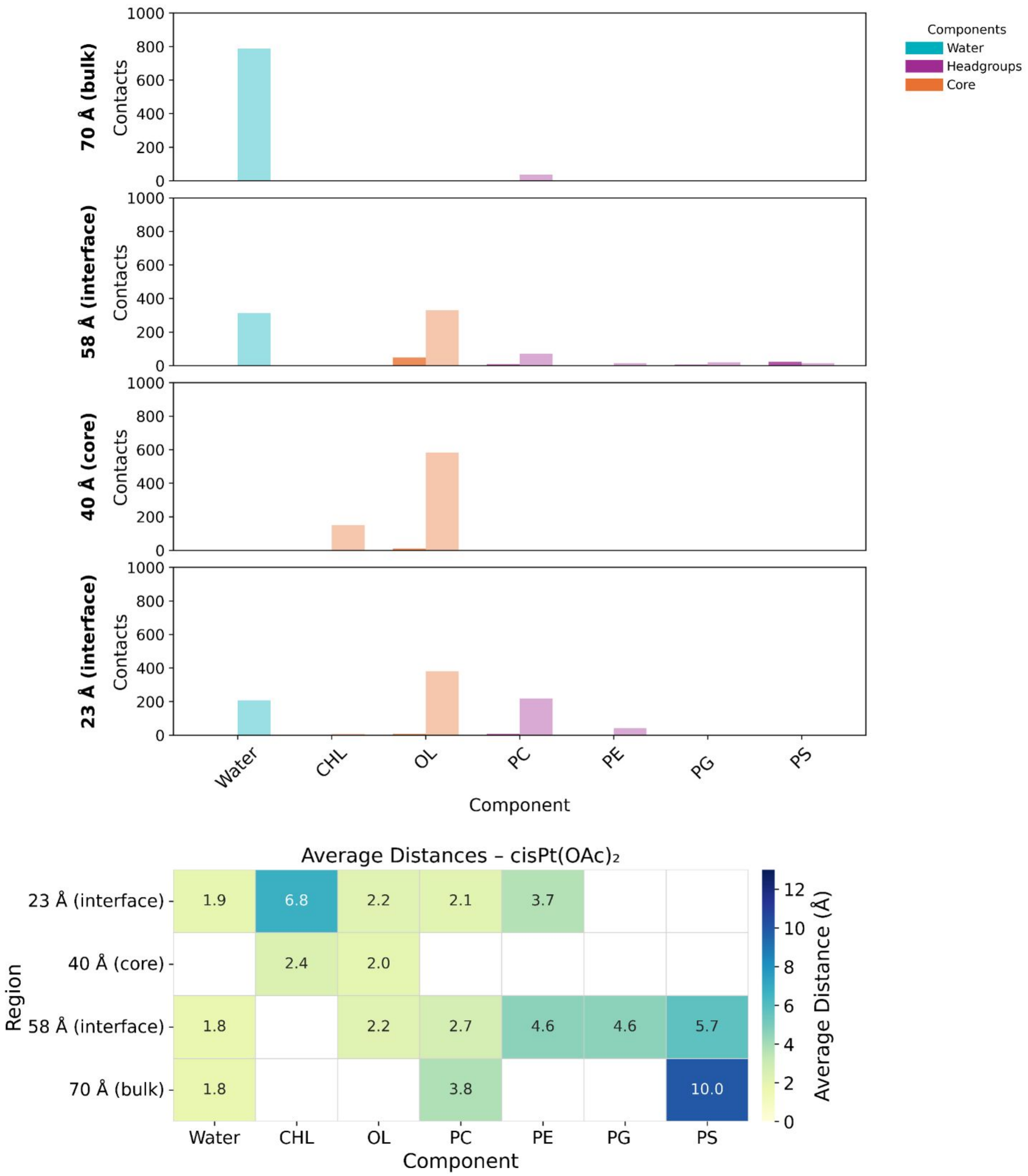

Figure S7. Hystogram plots of the contact analysis and the corresponding heatmap of the average distance between cisPt(OAc)$_2$ and the various groups at the four key positions, 70 Å (bulk), 58 and 23 Å (interfaces) and 40 Å (core).

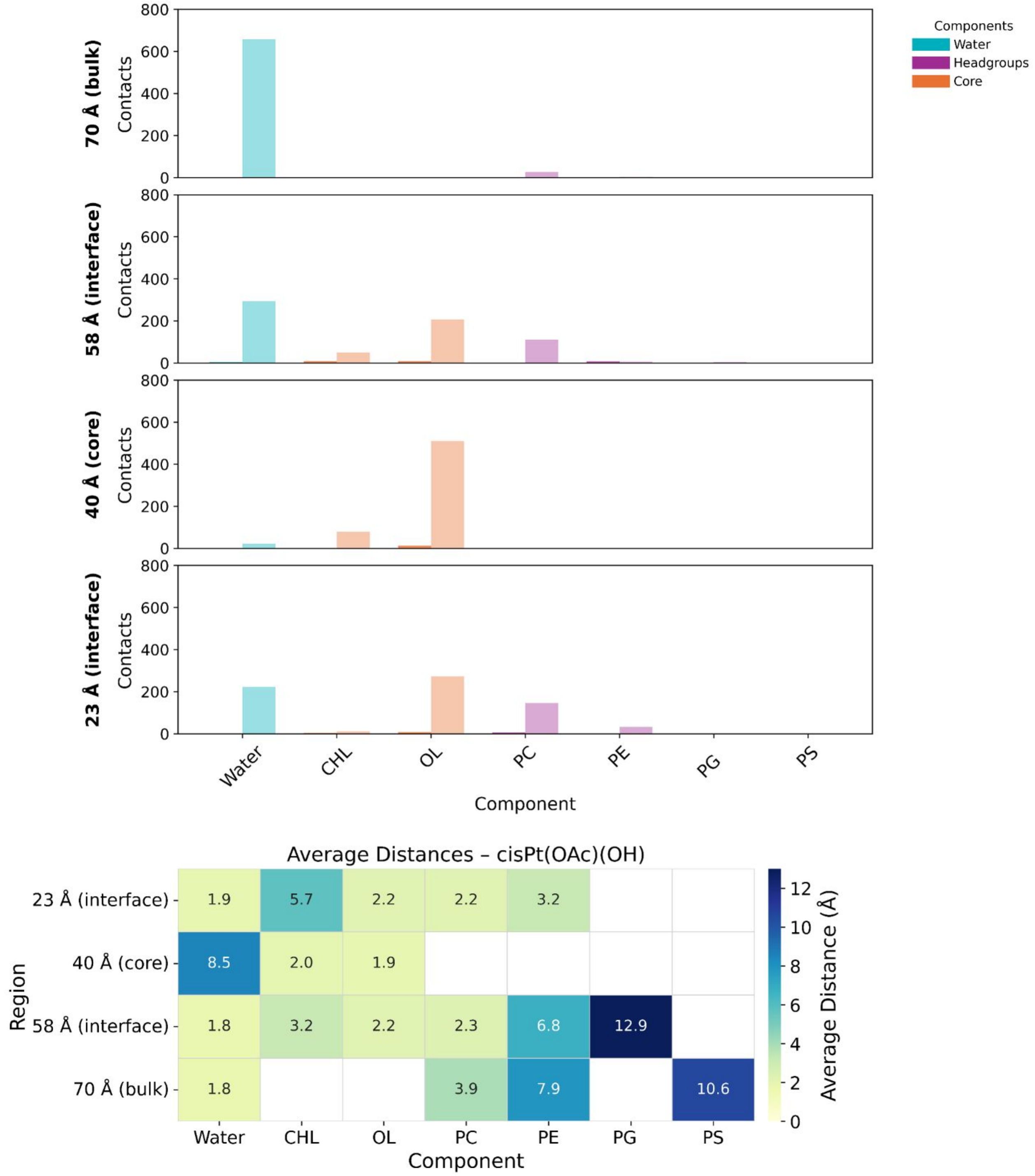

Figure S8. Histogram plots of the contact analysis and the corresponding heatmap of the average distance between cisPt(OAc)(OH) and the various groups at the four key positions, 70 Å (bulk), 58 and 23 Å (interfaces) and 40 Å (core).

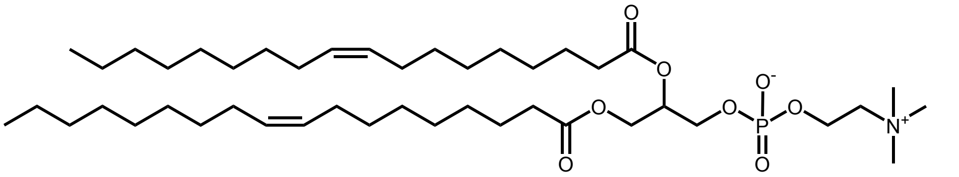

DOPC

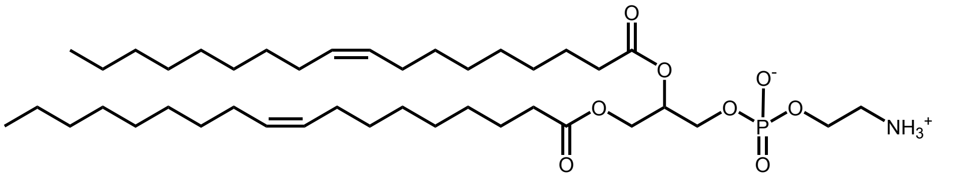

DOPE

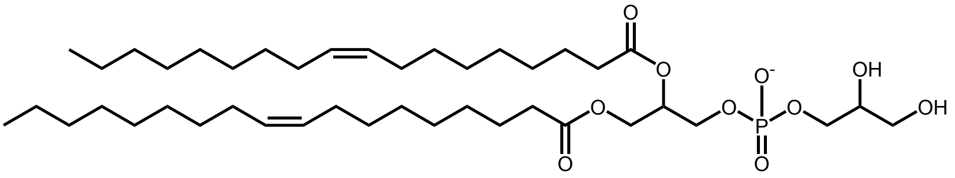

DOPG

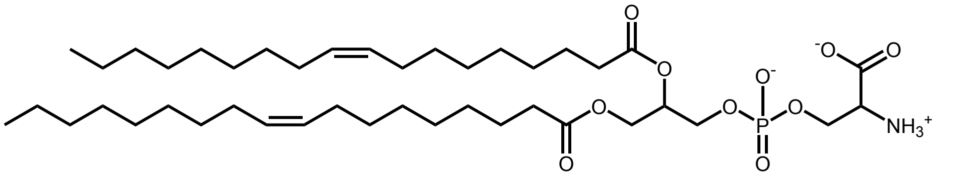

DOPS

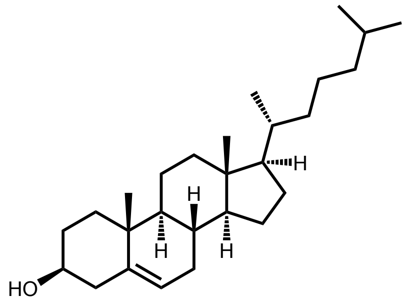

CHL

Figure S9. Chemical structure of the lipids that constitute the membrane model used in this work.

References

1. Roux, B. The calculation of the potential of mean force using computer simulations. *Comput Phys Commun* **91**, 275–282 (1995).
2. Souaille, M. & Roux, B. Extension to the weighted histogram analysis method: combining umbrella sampling with free energy calculations. *Comput Phys Commun* **135**, 40–57 (2001).
3. Hub, J. S., de Groot, B. L. & van der Spoel, D. g_wham—A Free Weighted Histogram Analysis Implementation Including Robust Error and Autocorrelation Estimates. *J Chem Theory Comput* **6**, 3713–3720 (2010).
